# Multi-region sequencing with spatial information enables accurate heterogeneity estimation and risk stratification in liver cancer

**DOI:** 10.1186/s13073-022-01143-6

**Published:** 2022-12-16

**Authors:** Chen Yang, Senquan Zhang, Zhuoan Cheng, Zhicheng Liu, Linmeng Zhang, Kai Jiang, Haigang Geng, Ruolan Qian, Jun Wang, Xiaowen Huang, Mo Chen, Zhe Li, Wenxin Qin, Qiang Xia, Xiaonan Kang, Cun Wang, Hualian Hang

**Affiliations:** 1 Department of Liver Surgery, Renji Hospital, Shanghai Jiao Tong University School of Medicine, Shanghai, China; 2 State Key Laboratory of Oncogenes and Related Genes, Shanghai Cancer Institute, Renji Hospital, Shanghai Jiao Tong University School of Medicine, Shanghai, China; 3 Hepatic Surgery Center, Tongji Hospital, Tongji Medical College, Huazhong University of Science and Technology, Wuhan, China; 4 Renji Biobank, Renji Hospital, Shanghai Jiao Tong University School of Medicine, Shanghai, China; 5 Department of Gastrointestinal Surgery, School of Medicine, Renji Hospital, Shanghai Jiao Tong University, Shanghai, China; 6 Key Laboratory of Gastroenterology and Hepatology, Division of Gastroenterology and Hepatology, Renji Hospital, Shanghai Jiao Tong University School of Medicine, Shanghai, China

**Keywords:** Hepatocellular carcinoma, Intra-tumor heterogeneity, Immunotherapy, Prognostication

## Abstract

**Background:**

Numerous studies have used multi-region sampling approaches to characterize intra-tumor heterogeneity (ITH) in hepatocellular carcinoma (HCC). However, conventional multi-region sampling strategies do not preserve the spatial details of samples, and thus, the potential influences of spatial distribution on patient-wise ITH (represents the overall heterogeneity level of the tumor in a given patient) have long been overlooked. Furthermore, gene-wise transcriptional ITH (represents the expression pattern of genes across different intra-tumor regions) in HCC is also under-explored, highlighting the need for a comprehensive investigation.

**Methods:**

To address the problem of spatial information loss, we propose a simple and easy-to-implement strategy called spatial localization sampling (SLS). We performed multi-region sampling and sequencing on 14 patients with HCC, collecting a total of 75 tumor samples with spatial information and molecular data. Normalized diversity score and integrated heterogeneity score (IHS) were then developed to measure patient-wise and gene-wise ITH, respectively.

**Results:**

A significant correlation between spatial and molecular heterogeneity was uncovered, implying that spatial distribution of sampling sites did influence ITH estimation in HCC. We demonstrated that the normalized diversity score had the ability to overcome sampling location bias and provide a more accurate estimation of patient-wise ITH. According to this metric, HCC tumors could be divided into two classes (low-ITH and high-ITH tumors) with significant differences in multiple biological properties. Through IHS analysis, we revealed a highly heterogenous immune microenvironment in HCC and identified some low-ITH checkpoint genes with immunotherapeutic potential. We also constructed a low-heterogeneity risk stratification (LHRS) signature based on the IHS results which could accurately predict the survival outcome of patients with HCC on a single tumor biopsy sample.

**Conclusions:**

This study provides new insights into the complex phenotypes of HCC and may serve as a guide for future studies in this field.

**Supplementary Information:**

The online version contains supplementary material available at 10.1186/s13073-022-01143-6.

## Background

Liver cancer is the sixth most common tumor and represents the fourth leading cause of cancer-related death worldwide [1]. Hepatocellular carcinoma (HCC) is the dominant histological form of liver cancer, accounting for ~ 90% of all liver cancer cases [1]. In the last decade, considerable progress has been made in treating patients with HCC, owing to the advent of new molecular targeted therapies as well as immunotherapies [2–5]. Despite this, there remain a large number of patients with HCC who receive these therapies experiencing unfavorable outcomes, which may be attributed to the existence of tumor heterogeneity [6, 7].

Three aspects of tumor heterogeneity have been reported in HCC, including interpatient heterogeneity (IPH), inter-tumor heterogeneity, and intra-tumor heterogeneity (ITH) [7]. IPH represents the difference in tumors between patients and has been extensively investigated in studies regarding molecular classifications [8]. Inter-tumor heterogeneity is observed between tumor nodules of the patients with multi-focal HCC. As both intrahepatic metastasis (IM) and multicentric occurrence (MO) can promote formation of multi-focal HCC, it is challenging to characterize this class of heterogeneity [9, 10]. Intra-tumor heterogeneity represents the difference between different regions within the same tumor nodule. In HCC, ITH has been systematically explored using multi-omics approaches, including genomics [11–13], transcriptomics [13, 14], epigenomics [12, 15], and proteomics [13, 16]. Recently, increased attention has been brought to the clinical significance of ITH. Higher ITH has been demonstrated to be associated with worse survival outcome and higher risk of metastasis and recurrence [7, 17]. Moreover, ITH has also been implicated as a crucial contributor to drug resistance [18, 19]. Overall, accurate quantification of ITH is of considerable clinical and research importance.

Conventional multi-region sampling strategies adopted by most studies do not record the spatial details of samples, and, thus, potential influence caused by sampling bias on the estimation of ITH remains largely unexplored. To address this concern, a spatial localization sampling (SLS) strategy was proposed in the present study. This strategy can record the coordinates of each sampling site in two-dimensional (2D) space. Through SLS, we revealed a significant relationship between spatial and molecular heterogeneity. The normalized diversity score was thus developed to minimize the sampling bias and provide a more reliable evaluation of patient-wise ITH in HCC. In addition, gene-wise ITH that represented the expression pattern of genes across multi-region samples was also characterized. We developed a computational approach to quantitatively measure this kind of heterogeneity, which enabled direct comparison of the degree of ITH between different features.

## Methods

### Patient samples

Seventy-five tumors and 21 matched adjacent liver samples were collected from 14 patients who underwent partial hepatectomy for primary HCC at Renji Hospital of Shanghai Jiao Tong University School of Medicine. This study was approved by the Ethics Committee of Renji Hospital (KY2021–114–B), and all enrolled patients have provided informed consents. Clinical information of included patients was retrieved from the hospital electronic medical record system. All the patients were older than 18 years at diagnosis, had histopathologically confirmed HCC, presented with solitary tumors, had no evidence for a history of other malignancies, and were treatment-naïve prior to the surgery. To prevent sample degradation, specimens were obtained immediately after their removal from the surgical field and all the subsequent manipulations were performed on ice. The tumors were carefully excised, washed, and cut in half along the longitudinal axis. Research tissues were then harvested from the newly exposed tumor surface using a scalpel or a 10-mm disposable skin biopsy punch. To avoid cross contamination, a fresh blade or punch was used for every tumor sector. Each sampling site was at least 1 cm away from others and areas with apparent necrosis, fibrosis, hemorrhage, and cystic changes were avoided to maximize tumor cellularity. Adjacent liver specimens were collected from 10 out of 14 patients, which were typically at least 2 cm away from the tumor edge. All the specimens were snap frozen using liquid nitrogen and stored at –80 °C before further application. Histologic slides were stained with hematoxylin and eosin (H&E) and scanned using the Aperio CS Scanscope (Aperio Technologies, CA, USA). These H&E slides were then carefully reviewed by two experienced pathologists to ensure that the selected regions contained more than 70% tumor content, according to criteria of previous studies [15, 20].

### Spatial coordinate acquisition

We designed a spatial localization sampling (SLS) strategy to obtain the exact spatial coordinates of each sampling site (Additional file 1: Figure S1A and S1B). This strategy needs two assistive devices (multi-color localization needles and right-angle ruler) and one software (GetData Graph Digitizer version 2.26). Multi-color localization needles are used to mark the sampling sites; different colors of the needles indicate different sample numbers (for example, red needle represents sample #1 while yellow needle represents sample #10). A right-angle ruler is utilized to provide a fixed coordinate system across different tumors. After the given tumor was spatially organized with localization needles and right-angle ruler, we photographed it and imported the image into GetData Graph Digitizer software for coordinate extraction. This software can reconstruct a two-dimensional coordinate system without influence caused by perspective transformed image, thereby outputting comparable coordinates across different conditions. Notably, the SLS strategy only discerns coordinates in the *x–y* plane, and therefore, tumor sampling should be performed in the same *z*-plane to minimize height differences.

### Spatial transcriptomic/genomic analysis

We developed two approaches to correlate molecular data with spatial data. The first approach focused on calculating the pairwise physical/molecular distance between any two samples within a given tumor. Pairwise physical distance was calculated directly based on the coordinates of two sampling sites. As for the molecular distance, pairwise transcriptomic distance was measured using Spearman correlation, while pairwise genomic distance was determined based on Jaccard index [21]. Correlation analysis between physical and molecular distance was then conducted separately in each individual.

The second approach focused on investigating an overall correlation between physical and molecular diversity across all the tumors. Physical diversity represents a mean value of the distance from each sampling point to the center point; larger physical diversity indicated increased distribution of the sampling points. A previous computational strategy of the estimation of ITH was used as a reference to calculate the transcriptomic diversity [22]. In specific, principal component analysis (PCA) was performed on all tumor samples to extract the first 15 (according to the eigenvalues) principal components (PCs) from the original expression data. Transcriptomic diversity was then calculated separately in each tumor as follows:

$$\mathrm{Div}\left(t\right)=\frac{1}{m}{\sum }_{i=1}^{m}\sqrt{{\sum }_{j=1}^{n}{\left({x}_{ij}-{\mu }_{j}\right)}^{2}}$$

where *m* and *n* represented the number of multi-regional samples and the numbers of PCs, and *μ*_*j*_ represented the arithmetic mean of PCs across multi-regional samples. Higher transcriptomic diversity score indicates greater patient-wise transcriptomic ITH. As for the genomic diversity, according to a previous publication, it was defined as the median value of all pairwise genomic distance values within a given tumor [21]. Correlation analysis between physical and molecular diversity was then carried out on all individual tumors (14 for transcriptomic diversity and 10 for genomic diversity). Notably, since WES was only performed on three tumors (T10, T13, and T18), genomic diversity scores were calculated using data from RNA-based mutation calling.

### Patient-wise ITH quantification

In this study, we mainly focused on patient-wise ITH quantification at transcriptome level. The reliability of two previously published methods that estimated ITH using single-region sample, including a bulk sequencing-based method [23] and a single-cell sequencing-based method [22], was assessed in multi-region sequencing cohorts. The bulk sequencing-based method, which was implemented through DEPTH R package, was applied to the 75 multi-regional samples. Median value across all the samples was used to classify samples into high- and low-ITH groups. If all regions from a given tumor were classified into the low-ITH group, then this tumor was designated as uniformly ITH low; if all regions from a given tumor were classified into high-ITH group, then this tumor was designated as uniformly ITH high. If some regions from the same tumor were ITH high while others were ITH low, then this tumor was considered a discordant one. Single-cell sequencing-based method was applied to a cohort with two patients and seven samples following the instructions of a previous publication [22]. For statistical comparison, we randomly extracted 500 tumor cells each time from a given sample to calculate the single-cell diversity score, which was repeated 1000 times to determine the distribution of the diversity score of this sample. Then, comparison within the same tumor was conducted to explore whether single-cell sequencing-based ITH quantification was consistent across different tumor regions.

To minimize the potential influence of spatial distribution of sampling sites on ITH estimation, a normalized diversity score was proposed, which was calculated as follows:

$$\mathrm{Normalized diversity score}=\frac{Div\left(t\right)}{Div\left(p\right)}$$

where div (*t*) represented the transcriptomic diversity score and div (*p*) represented the physical diversity score.

Based on two tumors with largest sample size (T13 and T18), we performed a simulation to test the performance of the normalized diversity score. Two metrics, coefficient of variation (CV) and coefficient of deviation (CD), were adopted to investigate whether normalized diversity score could measure ITH more accurately. The calculation of CV was given as:

$$\mathrm{CV}=\frac{\sqrt{\frac{1}{N}{\sum }_{i=1}^{N}{\left({x}_{i}-\mu \right)}^{2}}}{\mu }$$

where *μ* represented the mean value. Higher CV indicates the greater level of data dispersion around the mean. We also proposed CD as a measurement of deviation from the “gold standard” diversity score (calculated based on all regions within a tumor). CD was defined as follows:

$$\mathrm{CD}=\frac{\sqrt{\frac{1}{N}{\sum }_{i=1}^{N}{\left({x}_{i}-\alpha \right)}^{2}}}{\mu }$$

where *α* represented “gold standard” diversity score. Higher CD indicates the greater level of data deviation from the “gold standard.”

### Gene-wise ITH quantification

For measuring gene-wise ITH level, we developed a computational strategy to calculate the integrated heterogeneity score (IHS). This strategy was based on two different approaches, variance-based and clustering-based approaches, which could complement each other to ensure reliable estimation of ITH. Variance-based approach calculated the intra-tumor variance (W = variance of differences within a tumor) and inter-tumor variance (B = variance of differences between patients) using linear mixed-effects analyses based on nlme R package [24, 25]. Intra-tumor variability score (ITVS) was then defined as follows:

$$\mathrm{ITVS}=\frac{W}{W+B}$$

Lower ITVS is associated with decreased ITH level.

Clustering-based approach was based on the concept that a gene with low ITH should have the ability to concordantly cluster samples derived from the same patients [26, 27]. We used hclust function in R to cluster samples into sequentially increased groups, from 1 to total number of patients. Patient group overall ratio (PGOR) was then calculated as follows:

$$\mathrm{PGOR}=\frac{N\left(\mathrm{patients.grouped.in.the.same.clusters}\right)}{N (\mathrm{total.number.of.patients})}$$

A curve based on PGOR could be obtained and the AUC of PGOR curve was calculated using numerical integration. Clustering concordance score (CCS) was then given as:

$$\mathrm{CCS}=1-\frac{\mathrm{AUC }(\mathrm{PGOR})}{N \left(\mathrm{total.number.of.patients}\right)-1}$$

IHS was determined on the geometric mean of ITVS and CCS, ranging from 0 to 1. A low IHS is associated with low gene-wise ITH level. According to IHS, we empirically divided genes into four groups, including (1) low-ITH group (0 ~ 0.25 IHS), (2) median-ITH group (0.25 ~ 0.50 IHS), (3) high-ITH group (0.50 ~ 0.75 IHS), and (4) very high-ITH group (0.75 ~ 1.00 IHS). Notably, in addition to the application of calculating gene-wise ITH, IHS strategy can also be applied to estimating ITH of other features, including immune infiltration.

### Public prognostic assessment cohorts

We collected six HCC cohorts with available survival information, including four sequencing-based cohorts (CHCC-HBV [28], LICA-FR [29], LIRI-JP [30], and TCGA-LIHC [31]) and two microarray-based cohort (GSE14520 [32] and GSE54236 [33]), which incorporated a total of 1189 HCC patients. Among the sequencing-based cohorts, LICA, LIRI, and LIHC provided raw counts, which were converted to TPM values for subsequent analyses [34], while CHCC only provided fragments per kilobase per million reads (FPKM) normalized data, which was also transformed into TPM values. For microarray-based cohorts, normalized data was directly downloaded from the Gene Expression Omnibus (GEO) database (www.ncbi.nlm.nih.gov/geo/). The survival data of LIRI cohort was downloaded from the International Cancer Genome Consortium (ICGC) portal (https://dcc.icgc.org/), data of LIHC cohort was achieved from TCGA Pan-Cancer Clinical Data Resource (TCGA-CDR) [35], and data of CHCC and LICA were obtained from the supplementary files of corresponding publications [28, 29]. Survival information of microarray-based cohorts were obtained from GEO database or corresponding publications.

### Public multi-region/focal cohorts

We collected four multi-region HCC cohorts with available expression profiles, including two sequencing-based cohorts (E-MTAB-5905 [14] and GSE136711 [36]) and two microarray-based cohorts (GSE56140 [37] and GSE92528 [38]), which included 43 HCC patients with a total of 139 multi-regional tumors. Raw sequencing data of E-MTAB-5905 was downloaded from the European Nucleotide Archive (ENA) database (www.ebi.ac.uk/ena). Sequencing reads were mapped to the hg38 reference genome using HISAT2 (version 2.2.1) [39]. Gene-level counts were then calculated using the subRead package [40]. The resultant count data was normalized using TPM for subsequent analyses. Sample H9.c was excluded from the downstream analyses since it was obtained from another tumor nodule of patient P09. Technical replicate of sample H2.a was also removed. The expression profiles of GSE136711 [36] were downloaded from the GEO database in the form of raw fragment counts, which were also normalized to TPM. For the two microarray-based cohorts, normalized expression profiles were directly obtained from the GEO database. Multi-region cohorts of solid tumors beyond HCC, including cervical cancer (GSE5787) [24], breast cancer (GSE23593) [41], lung cancer (GSE33532) [42], and high-grade glioma (GSE62802) [43], were also collected from the GEO database. Expression profiles of these cohorts were based on microarray; corresponding normalized data were thus directly downloaded for downstream analyses. Aside from multi-region expression cohorts, we also included a multi-focal HCC cohort GSE98617, which included 16 HCC patients with 36 multinodular tumors [9]. Normalized expression data was also obtained from GEO database.

### Single-cell RNA-seq analysis

A multi-region single-cell RNA-seq cohort GSE112271 was included, comprising two patients and seven tumor sectors [14]. Preprocessed expression data from CellRanger was downloaded from the GEO database. We utilized *Read10X* and *CreateSeuratObject* function in Seurat package to transform the raw Gene-barcode count matrix into Seurat object [44]. Tumor cells were discerned by a recently published algorithm named copy number karyotyping of aneuploid tumors (CopyKAT) [45]. Tumor cells in different tumor sectors were predicted separately, and the inferred tumor cells were extracted from the original Seurat object for downstream analysis.

### Statistical analysis

Statistical analyses and graphical visualization were performed in R software version 4.0.5 (https://cran.r-project.org/). Correlation between two continuous variables was determined by Pearson’s *r* correlation or Spearman’s rank-order correlation analysis. Comparison between continuous variables was performed using Student’s *t* test or Wilcoxon rank-sum test. Contingency table variables were analyzed by Fisher’s exact test. Hazard ratio (HR) was estimated using Cox regression model in survival R package [46]. Time-dependent AUC was computed using the timeROC R package [47]. Unless indicated otherwise, a two-tailed *P* < 0.05 was considered statistically significant. Detailed methods are presented in the Additional file 2: Supplementary Methods.

## Results

### Tumor sampling with location information

**Fig. 1:**
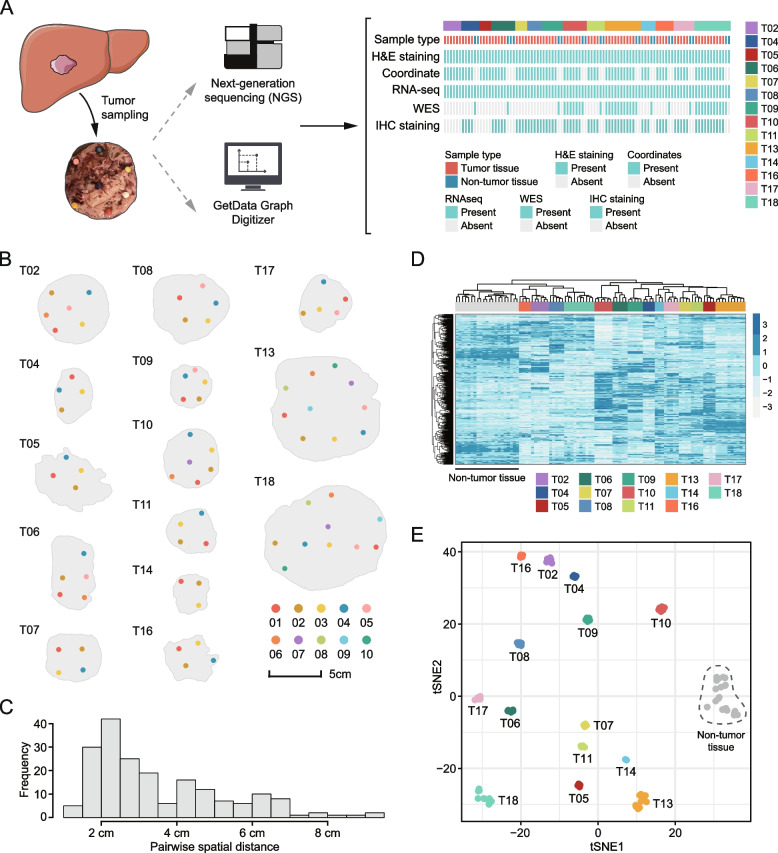
Multi-region sampling with spatial details. **A** Schematic presentation of sampling and summary of included samples. **B** Spatial distribution of multi-regional samples in each patient. These plots were transformed from the actual images, and thus the distributions in these plots were in perfect agreement with that in real cases. **C** The distribution of the pairwise spatial distance between sampling sites. Distance is provided in centimeters (cm). **D** The result of hierarchical clustering of all included samples (including 75 tumor and 21 non-tumor samples) based on the top 500 most variant genes. **E** Two-dimensional t-SNE plot of all samples based on PCA results

A total of 96 samples were obtained from 14 patients with HCC, including 75 tumor and 21 adjacent non-tumor liver samples. The number of tumor samples from each patient ranged from 3 to 10, with a median tumor number of 5. Detailed clinical information of included patients is presented in Additional file 3: Table S1. Samples were subjected to RNA sequencing (RNA-seq) at a depth of 40 M paired-end reads. Whole-exome sequencing (WES) was performed on 36 samples from patients T10, T13, and T18 (*n* = 26) as well as adjacent non-tumor tissues (*n* = 10) with an average of 100X depth. The spatial position of each sampling site was marked using SLS strategy (Additional file 1: Figure S1A and S1B) and schematic representation of the geographic distribution of tumor sectors in each individual is shown in Fig. 1B. 2D spatial coordinates (*x* and *y*) of tumor sectors were also obtained via SLS (Additional file 3: Table S2). Pairwise spatial distance between any two sectors ranged from 1.07 to 9.43 cm, with a median value of 2.80 cm (Fig. 1C).

We performed hierarchical clustering using the top 500 most variant genes based on the 96 samples and found that tumor samples were clustered by patient perfectly, indicating a greater IPH than ITH (Fig. 1D). Besides, t-distributed stochastic neighbor embedding (t-SNE) analysis was performed to visualize samples in a scatter plot (Fig. 1E). Clear separations between tumor samples derived from different patients could also be observed in the t-SNE result. Non-tumor samples tended to cluster together independent of the patient source, which was consistent with findings from single-cell studies [22, 48, 49].

### A positive correlation exists between spatial and molecular heterogeneity

**Fig. 2:**
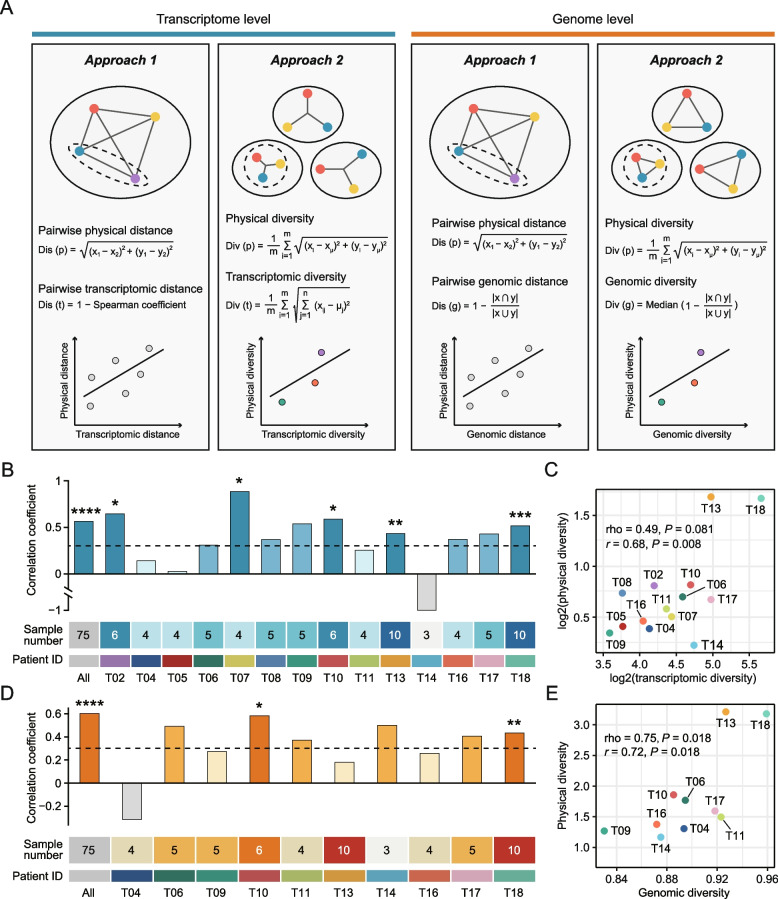
Correlation between spatial and molecular heterogeneity. **A** Schematic illustration of the distance-based and diversity-based approaches. **B** Spearman correlation between transcriptomic and physical distance in each individual. **C** Correlation between transcriptomic and physical diversity across all tumors. **D** Spearman correlation between genomic and physical distance in each individual. **E** Correlation between genomic and physical diversity across all tumors. *P < 0.05, **P < 0.01, ***P < 0.001, ****P < 0.0001

The relationship between molecular and spatial data was explored using distance-based and diversity-based approaches (see details in Additional file 2: Supplementary Methods) (Fig. 2A). Analyses at the transcriptome level were first conducted. A positive correlation between pairwise physical and transcriptomic distance could be observed in 13 out of 14 tumors, and the correlation coefficients were larger than 0.30 in 10 out of 13 tumors, suggesting a clear positive trend (Fig. 2B and Additional file 1: Figure S2). Importantly, this correlation was also significant and remarkable at the overall level across all tumors (Spearman rho = 0.56, *P* < 0.001) (Fig. 2B). In the other approach, transcriptomic diversity, an indicator of patient-wise ITH, was calculated (Fig. 2A). A significant correlation determined by Pearson correlation analysis between physical and transcriptomic diversity scores was demonstrated (Fig. 2C). These results indicate that (1) higher physical distance between two sectors within a tumor is associated with greater transcriptomic difference; (2) more dispersed spatial distribution of sampling sites is associated with higher transcriptomic diversity.

Next, similar analyses at the genome level were performed. In three tumors (T10, T13, and T18) with available WES data, significant correlation between physical and genomic distance could be observed (Additional file 1: Figure S3A and 3B). For other tumors that were not subjected to WES, RNA-based mutation calling was conducted as a complement (Additional file 1: Figure S4A). The median number of RNA-derived mutations was 183 across 56 multi-region samples of 10 tumors (Additional file 1: Figure S4B). A positive correlation between pairwise physical and genomic distance could be observed in 9 out of 10 tumors, and the correlation coefficients were larger than 0.30 in 6 out of 9 tumors (Fig. 2D). At the overall level, this correlation was also remarkable (Spearman rho = 0.60, *P* < 0.001) (Fig. 2D). As expected, physical and genomic diversity scores were also found to be significantly correlated (Fig. 2E). These findings collectively indicate that the evaluation of ITH at the genome level can be influenced by the sampling bias as well.

### Normalized diversity score provides a more accurate estimation of patient-wise ITH than other metrics

Accurate quantification of patient-wise ITH has great significance in clinical and research fields. Many methods have been developed to measure ITH based on single-region tumor samples. Herein, two representative methods depending on single-region bulk and single-region single-cell sequencing were tested, respectively. The bulk sequencing-based method, DEPTH, was first evaluated on our cohort [23]. Samples were stratified into ITH-high and ITH-low groups according to the median DEPTH score. It could be observed that half of the tumors (7 out of 14 tumors) contained both high-ITH and low-ITH samples (Additional file 1: Figure S5A). Next, the single-cell-based method was evaluated on a single-cell cohort, which included 7 samples from 2 tumors (H13 and H14) (Additional file 3: Table S3) [14]. Single-cell diversity score of each sample was determined as described previously [22]. Unsurprisingly, the diversity scores across different intra-tumor regions varied greatly, both in tumors H13 and H14 (Additional file 1: Fig. S5B). In summary, these results indicate that single-region ITH estimation is susceptible to sampling bias and may poorly represent the actual ITH level.

**Fig. 3:**
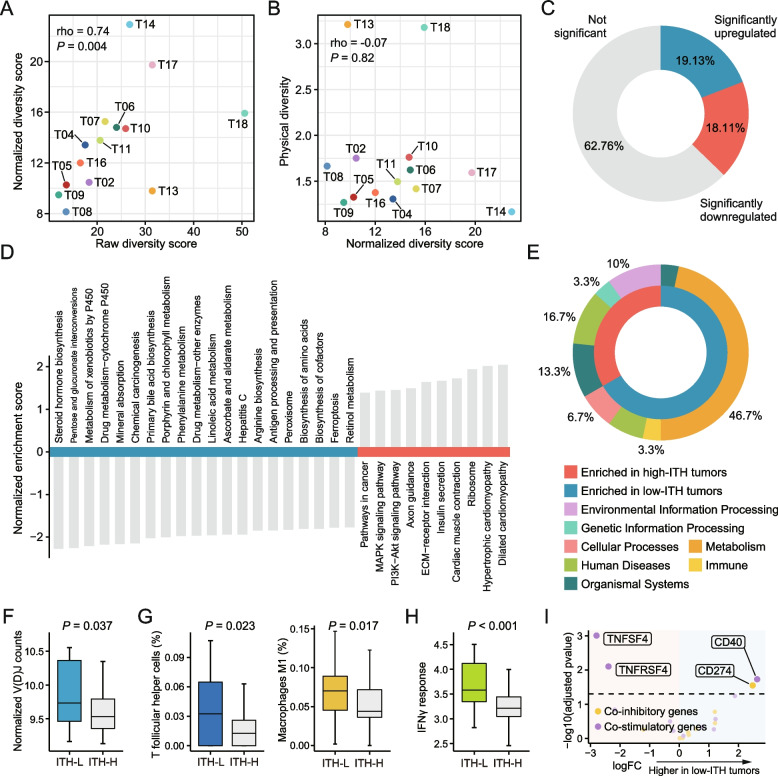
Characterization of low-ITH and high-ITH tumors. **A** Spearman correlation between normalized and raw diversity scores. **B** Spearman correlation between normalized diversity scores and physical diversity. **C** The proportion of differentially expressed genes (DEGs) between low-ITH and high-ITH tumors. **D** Differential biological processes between two classes based on KEGG enrichment analysis. **E** Clustering of enrichment results based on KEGG pathway metadata. **F** Difference of normalized V(D)J counts between two classes. Higher normalized V(D)J counts indicate more infiltration of tumor-infiltrating lymphocyte (TIL). **G** Difference of abundance of T follicular helper cells and M1 macrophages between two classes. **H** Difference of activity of IFN-γ response between two classes. Pathway activity was determined via calculating the mean value of expression of genes included in IFN-γ response pathway. **I** Heatmap showing the difference of VIPER-inferred protein activity of immune checkpoint genes between two classes. Statistical significance of difference was determined using Wilcoxon rank-sum test

Studies have reported the use of multi-region samples for ITH quantification [21, 50–54]. Although this approach minimizes potential bias caused by single-region sampling to some extent, it can still be influenced by spatial distribution of sampling sites, as stated above. Therefore, we proposed a normalized diversity score for quantifying ITH, which was calculated by dividing the transcriptomic diversity with the physical diversity. Residual analysis showed that the relationship between transcriptomic and physical diversity appeared to be linear, suggesting that the calculation of normalized diversity score was reasonable (Additional file 1: Figure S6A). Normalized diversity scores were still correlated to the raw ones (Fig. 3A) but were no longer affected by the spatial diversity, demonstrating that the sampling bias had been normalized out successfully (Fig. 3B). Higher normalized scores indicated a greater level of patient-wise ITH.

To demonstrate the superiority of the normalized diversity score in quantifying ITH, we performed sampling simulations in two tumors (T13 and T18) with the largest multi-regional sample size (*n* = 10). Suppose we could only randomly include five samples in each tumor; these samples were then used to calculate normalized and raw diversity scores. In this situation, diversity scores from a total of 252 random combinations were obtained (Additional file 1: Fig. S6B and 6C). In theory, a good metric should meet the following criteria: (1) it should have good representativeness (implying that the resultant scores should remain consistent across varying combinations); (2) it should have high accuracy (indicating that the resultant scores should be close to the “gold standard” score). Two metrics, CV (a metric of representativeness) and CD (a metric of accuracy), were used to evaluate the performance of normalized and raw diversity scores. Normalized diversity score consistently showed a lower CV than unnormalized ones in both tumors across different numbers of random sampling (*n* = 2 ~ 9), suggesting better representativeness of normalized score (Additional file 1: Figure S6D). As for the accuracy, we found that normalized scores tended to have a lower CD value when the random sampling number was small (*n* < 5) in both tumors (Additional file 1: Figure S6E). In the condition of using a large random sampling number (*n* ≥ 5), the CD differences between normalized and raw scores have become very small. It should be noted that a small sampling number (*n* < 5) was typically adopted by most of multi-region studies [14, 21, 37]. Accordingly, we consider that the normalized diversity score is an effective measurement approach for patient-wise ITH, especially in the cases with limited multi-region samples.

Line plots showing the relationship between CV/CD and sampling numbers were generated to further explore the appropriate sampling number for accurate estimation of ITH (Additional file 1: Figure S7A and S7B). A sharp decrease of CV/CD occurred when the sampling number was less than 3, indicating that a minimum of three samples might be required.

### Low-ITH tumors show enhanced activities in metabolism and immune pathways

The degree of ITH for each patient was determined based on the normalized diversity scores (Additional file 3: Table S4). The association between ITH and clinical features was first explored. There was a trend towards higher degree of ITH in patients with more advanced tumors or with higher serum concentration of alpha-fetoprotein (AFP), albeit the results did not achieve statistical significance (Additional file 1: Figure S8).

The 14 tumors were then assigned to low-ITH and high-ITH classes according to the median value. Comparison of expression profiles between two classes indicated that over one third of the genes were differentially expressed (5769 out of 15,490, 37.24%) (Fig. 3C; Additional file 3: Table S5). Enrichment analysis was performed on the results of differential expression analysis using KEGG gene sets (Fig. 3D; Additional file 3: Table S6). Intriguingly, nearly half of significant gene sets (46.7%, 14/30) were involved in metabolic processes, all of which were enriched in low-ITH tumors (Fig. 3E). On the contrary, tumor-associated pathways, such as MAPK signaling pathway and PI3K − Akt signaling pathway, tended to be related to high-ITH tumors (Fig. 3D).

Next, we sought to dissect the immune-associated characteristics of these two classes. Through calculating the normalized read counts mapped to V(D)J loci, the overall burden of tumor-infiltrating lymphocyte (TIL) in each bulk sample was estimated. The comparison between two classes showed that there was a higher overall burden of TIL in low-ITH tumors than in high-ITH ones (Fig. 3F). We further adopted the CIBERSORT algorithm to infer the relative abundance of 22 immune cell types, aiming to find out cell types showing differential infiltration between two classes [55]. As a result, four cell types, including memory resting CD4 T cells, T cells follicular helper (Tfh), M1 macrophages and neutrophils, were identified; of these, Tfh cells and M1 macrophages have higher infiltration in low-ITH tumors (Fig. 3G), while memory resting CD4 T cells and neutrophils are more abundant in high-ITH tumors (Additional file 1: Figure S9). Notably, higher infiltration of Tfh cells and M1 macrophages was found to be associated with increased inflammatory and antitumor immune response [56]. Given the close relationship between the interferon-γ (IFN-γ) signaling and antitumor immunity, comparison of activity of IFN-γ response signaling between two classes was then conducted. As expected, low-ITH tumors also exhibited higher activity of IFN-γ response than high-ITH tumors (Fig. 3H).

Above results prompted us to further examine whether the activity of immune checkpoint genes also differed between two classes. Compared with gene expression, protein activity represents a more reproducible biomarker and has greater potential to uncover therapeutic vulnerabilities [57]. The protein activity of 21 immune checkpoint genes (including 9 co-inhibitory and 12 co-stimulatory genes) was first inferred using a newly developed approach named weighted VIPER [58]. Comparison of checkpoint activity between two classes was then conducted, and the results showed that low-ITH tumors exhibited higher protein activity of CD40 and CD274 (PD-L1), while high-ITH tumors have higher activity of TNFSF4 (OX40L) and TNFRSF4 (OX40) (Fig. 3I). Based on this result, it can be speculated that patients with low-ITH tumors might be more suitable to be treated with PD-L1 inhibitor or OX40/OX40L agonist, while the patients with high-ITH tumors might be more likely to gain benefit from CD40 agonist.

### Gene-wise ITH can be determined by calculating IHS

**Fig. 4:**
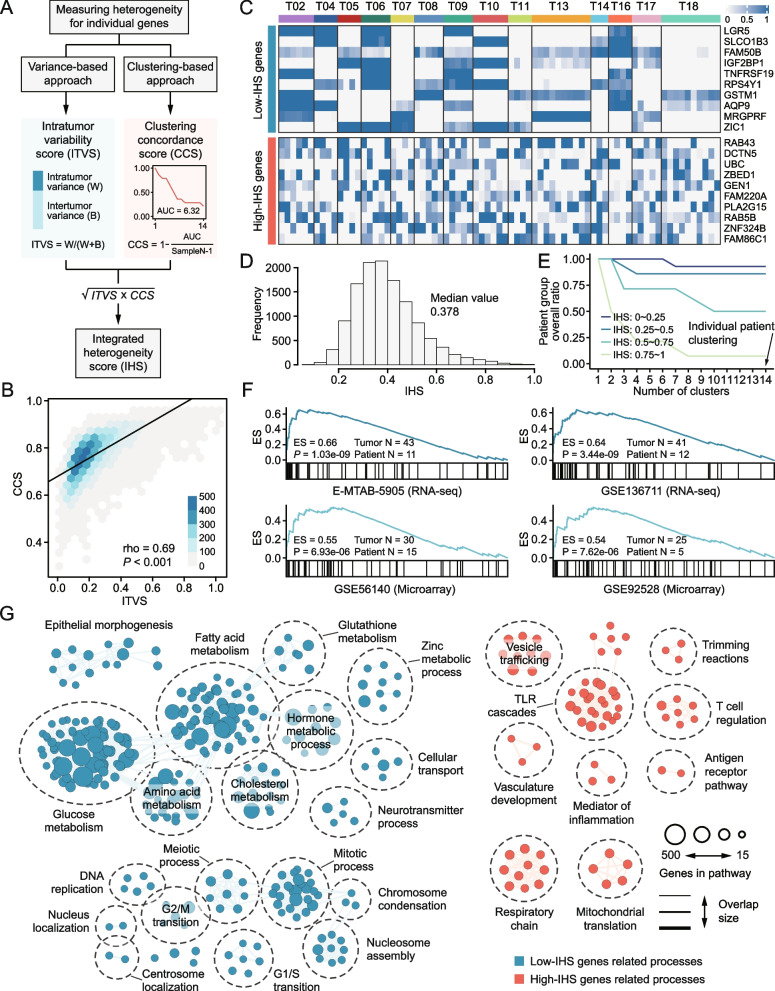
Calculation of gene-wise ITH. **A** Schematic presentation of the calculation processes of integrated heterogeneity score (IHS). **B** Spearman correlation between intra-tumor variability scores and clustering concordance scores. **C** Heatmap of the expression level of the top 10 genes with lowest and highest IHS. **D** The distribution of IHS of protein-coding genes. **E** Patient group overall ratio (PGOR) curves of genes with varying IHS. Lower PGOR indicates higher ITH. **F** Enrichment results of top 100 genes with lowest IHS of our cohort against ranked IHS results from other four public cohorts. **G** Enrichment map based on GSEA results. Blue nodes represent biological processes associated with low-ITH genes, while red nodes represent biological processes related to high-ITH genes. The thickness of edges is proportional to the overlap between the gene sets. Biological processes with similar function were grouped in cluster and labeled manually

Aside from patient-wise ITH, we also sought to determine the gene-wise ITH, which could be used to identify genes with low variation in expression across different intra-tumor regions. A computational strategy was developed to calculate the integrated heterogeneity score (IHS), an integrated metric based on intra-tumor variability score (ITVS) and clustering concordance score (CCS) (Fig. 4A; Additional file 3: Table S7). The result of correlation analysis indicated that ITVS and CCS were highly but not perfectly related, and thus, they might complement each other to achieve reliable estimation of gene-wise ITH (Fig. 4B). Expression patterns of the top 10 genes with lowest and highest IHSs across multi-regional samples are presented in Fig. 4C. It can be observed that low-IHS genes exhibit low variation in expression within the same tumor but may have high variation across different tumors (Fig. 4C). Distribution of IHSs of protein-coding genes was evaluated and the result showed the median value of IHS was 0.378 (Fig. 4D). To explore the distribution of random IHSs, we generated simulated expression data fitted with the negative binomial distribution. The median value of IHS calculated on simulated genes was 0.953 (Additional file 1: Figure S10A), which was significantly higher than that of real genes (Additional file 1: Figure S10B). According to IHS, genes could be classified into four groups, including low-ITH (0 ~ 0.25 IHS), median-ITH (0.25 ~ 0.50 IHS), high-ITH (0.50 ~ 0.75 IHS), and very high-ITH groups (0.75 ~ 1.00 IHS). Patient group overall ratio (PGOR) was calculated using genes from these four groups. As expected, genes in low-ITH group exhibited the highest ability to concordantly cluster patient samples (Fig. 4E).

As spatial data was not required for IHS analysis, IHS analysis was conducted using four additional public multi-region HCC cohorts for validation (Additional file 3: Table S8). The top 100 genes with lowest IHSs were first collected as a query gene set (low-ITH genes are more likely to have research implications). Gene set enrichment analysis (GSEA) was then performed using this gene set against normalized and ranked IHS results (from low to high) derived from the four public cohorts. Significant positive enrichment was observed in all cohorts, demonstrating that the IHS results are not cohort-dependent and can be generalized to other HCC cases (Fig. 4F).

To determine whether gene-wise ITH was tumor type-specific, we also collected multi-region clinical cohorts of four other tumor types, including cervical cancer, breast cancer, lung cancer and high-grade glioma, and calculated the IHSs of all genes for each tumor type. Similar to the analysis above, we examined the enrichment of low-IHS genes from HCC in other four tumor types. Interestingly, there was no significant enrichment across all these tumor types, suggesting that the gene-wise ITH may have certain tumor specificity (Additional file 1: Figure S11).

### Immune features show high variation across spatially distinct regions

Enrichment map was generated to delineate the relationship between gene-wise ITH and biological processes. The results revealed that low-ITH genes were associated with metabolism and cell cycle-related processes (such as fatty acid metabolism, amino acid metabolism, and G2/M transition), while high-ITH genes tended to be related to immune-related processes (such as T cell regulation and antigen receptor pathway) (Fig. 4G). Considering that the heterogeneity of immune features in HCC remains under-explored, further investigations regarding this aspect were thus carried out [13, 21, 36].

**Fig. 5:**
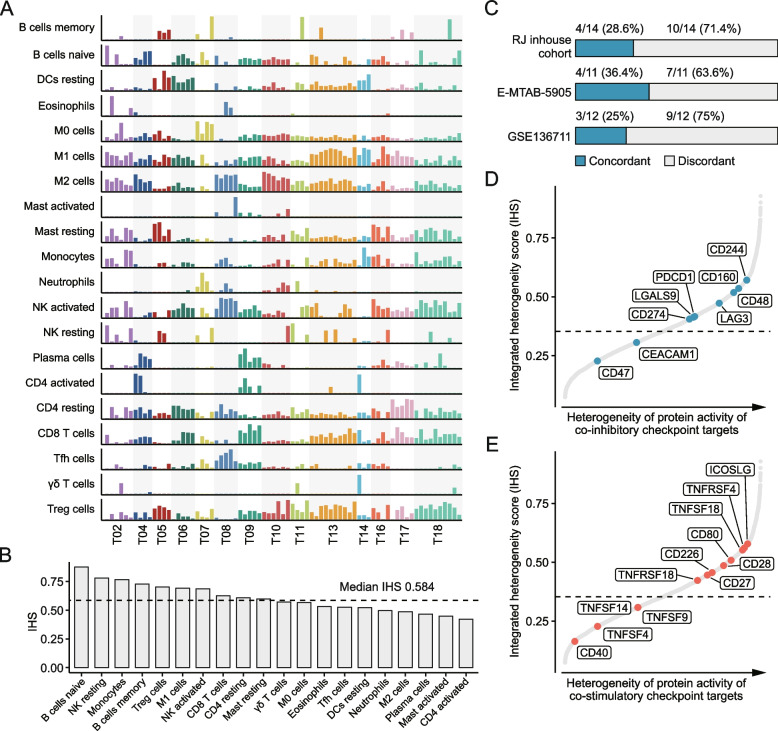
Immune heterogeneity in HCC. **A** The proportion of 22 CIBERSORT-based immune cell types across 75 multi-regional tumor samples. **B** IHS of 22 estimated cell types. **C** The proportion of tumors that have concordant or discordant immune profiles across different intra-tumor regions in three multi-region sequencing cohorts. **D** IHS of protein activity of 9 co-inhibitory immune checkpoint genes. **E** IHS of protein activity of 12 co-stimulatory immune checkpoint genes. Dotted lines indicate the median IHS value across 5099 proteins

Distribution of CIBERSORT-based 22 immune cell types across 75 multi-region samples was first analyzed (Fig. 5A). To quantitatively evaluate the immune cell heterogeneity, IHS for each cell type was calculated; the median IHS of 22 cell types was 0.584, which was numerically higher than that of protein-coding genes (IHS = 0.378) (Fig. 5B). Interestingly, we found that B cell seemed to be the most heterogenous cell type within tumors, with the IHS of over 0.7 (Fig. 5B). To determine whether this phenomenon is specific to HCC, we calculated the IHS of 22 immune cell types in other four solid tumor types mentioned above (Additional file 1: Figure S12A). These results were further integrated by the Robust Rank Aggregation (RRA) method [59]. The integrated result revealed that B cell was also one of the most heterogenous cell types across the four tumor types, implicating that this phenomenon might not be specific to HCC (Additional file 1: Figure S12B). Then, we collected 141 immune genes from previous publication to generate an immune gene expression profile [60]. In line with findings in enrichment analysis, immune genes showed significantly higher IHSs than other genes (Additional file 1: Figure S13A). Mahalanobis distance was calculated on this immune profile to determine the immune similarities of different intra-tumor regions (Additional file 1: Figure S13B-D). Tumors with outlier samples were considered to have discordant intra-tumor immune profiles, according to the previously defined criteria [36]. It could be observed that most of the tumors exhibited discordant immune profiles in all the three cohorts, indicating an overall high heterogeneity of immune gene expression (Fig. 5C). As a complement to computational analyses, immunohistochemistry (IHC) staining for CD45 was also performed to assess the immune cell heterogeneity. Images of a representative tumor are presented in Additional file 1: Figure S14A. IHS analysis showed a highly variable number of CD45^+^ cells across different intra-tumor regions (IHS = 0.799) (Additional file 1: Figure S14B). Collectively, above findings suggest that HCC might have a highly heterogenous immune microenvironment, and, thus, single-region-based evaluation of immune status of HCC might not be reliable.

We further sought to depict the heterogeneity of protein activity of immune checkpoint genes within tumors. Determining this kind of heterogeneity would have substantial clinical implications. For example, immuno-oncology agents targeting high-ITH checkpoint genes might lead to variation in treatment efficacy across different regions within tumors; such variation in efficacy enables some tumor cells survive during the treatment, and thus may facilitate the development of acquired resistance [6]. Accordingly, a checkpoint gene with low ITH could have greater therapeutic potential. We calculated the IHSs of VIPER-inferred protein activity of 9 inhibitory and 12 stimulatory checkpoint genes and found that an inhibitory checkpoint gene CD47 (IHS: 0.228) and a stimulatory checkpoint gene CD40 (IHS: 0.164) have relatively low ITH in protein activity among the tested genes (Fig. 5D and 5E). Both CD47 and CD40 have corresponding inhibitors or agonists being tested clinically [61, 62]. Our findings might provide a theoretical rationale for treating HCC with these agents.

### LHRS has an excellent power to prognosticate survival in HCC patients and is applicable to single-region tumor samples

Aiming to interrogate whether IHS was related to prognostic significance, six public cohorts with a total of 1189 HCC were utilized for prognostic analysis (Additional file 3: Table S8). Prognosis-associated genes (*P* < 0.05) were classified into four groups according to IHS, and the proportions of prognostic genes in each group for each HCC cohort were determined. There was an obvious trend that genes with lower IHS, which were calculated on all three multi-region sequencing cohorts, were more likely to have prognostic relevance (Additional file 1: Figure S15). Accordingly, low-ITH genes could be more informative for prognostication. Given that almost all previously reported prognostic signatures for HCC did not consider potential influences caused by ITH, developing a signature that can overcome ITH bias has significant clinical implication in HCC.

**Fig. 6:**
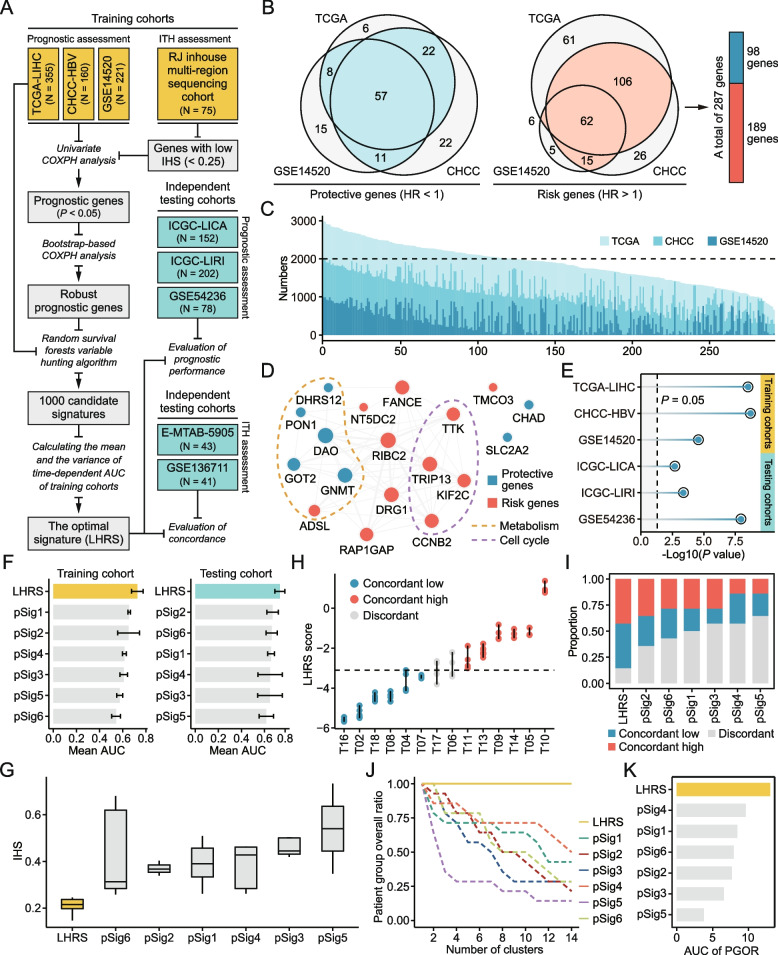
Construction and validation of LHRS. **A** Flow chart of the construction and validation of prognostic signature for HCC. **B** The overlap of the results of univariate Cox regression analysis across three training cohorts. Protective and risk genes were identified, respectively. **C** The numbers of iterations with significant results (*P* < 0.05) for 287 genes from preliminary screening. **D** Functional similarity of genes in LHRS. Functional annotation was added based on the information from KEGG pathway metadata. **E** The results of univariate Cox regression analysis of LHRS across training and testing cohorts. **F** Comparison of mean time-dependent AUC values between LHRS and six previously published signatures in training and testing cohorts. **G** Comparison of IHS of signature genes between LHRS and other six published signatures. **H** The distribution of LHRS scores of 75 multi-regional tumor samples. Each point indicates a single region and the vertical lines represent the distribution range of LHRS scores for each individual. All samples were divided into low-ITH and high-ITH groups based on the median value. Blue nodes represent the tumors with all regions classified into the low-ITH group; red nodes represent the tumors with all regions classified into the high-ITH group; grey nodes represent the tumors with both low-ITH and high-ITH regions. **I** Comparison of the percentages of patients who were classified as concordant low risk (blue), concordant high risk (red) or discordant (gray) between LHRS and other six published signatures. A lower discordant proportion indicates a better performance. **J** Patient group overall ratio (PGOR) curves of LHRS (solid line) and other six signatures (dashed line). **K** Comparison of AUC values of PGOR curves between LHRS and other six signatures

A multi-step strategy was proposed to construct the optimal prognostic signature (Fig. 6A). Specifically, univariate Cox proportional hazards regression (COXPH) analysis was first performed based on 1275 low-ITH genes (IHS < 0.25) in three training cohorts. This preliminary screening yielded 98 protective genes (HR < 1) and 189 risk genes (HR > 1) (Fig. 6B). These genes were further screened by a bootstrapping-based approach to determine prognostic markers with superior reproducibility. A total of 121 genes stably associated with patient survival were identified in over 2000 iterations (Fig. 6C). Random survival forest (RSF) analysis was then performed to determine the most significant markers. RSF was performed with 1000 replicates and the gene combination with the best performance was designated as the low-heterogeneity risk stratification (LHRS) signature. The LHRS comprised 18 genes, and most of these genes (11/18) were risk genes (Fig. 6D; Additional file 3: Table S9).

A good signature for prognostication should meet the following criteria: (1) it should give an accurate prediction of patient survival; (2) it should maintain uniform measurements across different intra-tumor regions. A comprehensive evaluation of LHRS was thus performed regarding these two aspects. Prognostic performance of LHRS was first assessed using public HCC cohorts. Univariate COXPH and log-rank analyses both indicated that LHRS was a significant prognostic factor (Fig. 6E and Additional file 1: Figure S16A-B). After adjusting for gender, age, and clinical stages using multivariate analysis, LHRS remained significantly associated with prognosis, implying that it was an independent prognostic factor (Additional file 1: Figure S17). In addition, six published HCC signatures, pSig1-6 [33, 63–67], were retrieved from previous publications for comparisons (Additional file 3: Table S10). Time-dependent area under the receiver operating characteristic curve (AUC) values were used to determine predictive performance. LHRS exhibited an overall better performance compared with other signatures both in training cohorts and independent testing cohorts (Fig. 6F and Additional file 1: Figure S16C-D).

Subsequently, ITH of the LHRS signature was evaluated based on the multi-region sequencing data. Three different metrics were adopted to explore whether LHRS was affected by ITH. Comparison of the median IHS of signature genes was conducted; genes in LHRS had the lowest median IHS value (0.216) (Fig. 6G). The proportion of discordant tumors was calculated; the discordant proportion of LHRS was lower than that of other signatures (Fig. 6H and I). PGOR analysis was also conducted to evaluate the ability of signatures to concordantly cluster tumors (Fig. 6J). Not surprisingly, LHRS presented higher PGOR-based AUC values compared with that of the other signatures (Fig. 6K). These findings were further validated using two independent multi-region HCC cohorts, E-MTAB-5905 (Additional file 1: Figure S18A, S18C and S18E) and GSE136711 (Additional file 1: Figure S18B, S18D and S18F). LHRS still exhibited an impressive performance in validation cohorts, although it might not always be the best one. Overall, above findings demonstrate that LHRS can provide an accurate prediction of outcome for patients with HCC based on single-region tumor samples.

### LHRS cannot be generalized to patients with multi-focal HCC

A significant number of HCC patients are diagnosed with multiple tumor nodules [68]. A multi-focal HCC cohort GSE98617, including 36 multinodular tumors from 16 HCC patients, was thus obtained and analyzed to explore whether LHRS was still applicable in those patients. IHS for each gene were first calculated using the multi-focal microarray data. The results showed a median IHS value of 0.799, which was considerably higher than that of other multi-region cohorts (Additional file 1: Figure S19A). Similar analyses as above were conducted subsequently. LHRS had a relatively high median IHS (0.715) with a low ranking across all signatures (6th/7) (Additional file 1: Figure S19B). The proportion of discordant tumors calculated based on LHRS also ranked poorly (6th/7), with approximately half of tumors (43.8%, 7/16) classified as discordant ones (Additional file 1: Figure S19C). Moreover, despite a high ranking (2nd/7), the PGOR-based AUC value of LHRS (AUC = 6.12) was still quite low relative to that of a perfect theoretical signature (AUC = 15) (Additional file 1: Figure S19D). These findings indicate that LHRS may not be a suitable signature for predicting overall survival of patients with multi-focal HCC, possibly owing to the high degree of inter-tumor heterogeneity.

## Discussion

ITH in HCC has been well characterized by numerous studies [12–14, 21, 69]. However, these studies typically did not collect spatial information of samples during the sampling process. Recently developed spatial transcriptomic methods offer a feasible solution to the problem of spatial information loss; however, they are only designed to explore cellular-scale (micro-level) spatial heterogeneity [70]. Therefore, approaches for studying heterogeneity at the level of the entire tumor (macro-level) remain absent. A previous study on melanoma has made an attempt to record spatial data of multi-region sampling [71]. However, the approach used in the study only generated approximate spatial information without exact coordinates, limiting the possibilities for downstream quantitative analysis [71]. Herein, a simple and easy-to-implement strategy named SLS was presented to circumvent this limitation, which could obtain exact coordinates of sampling sites by image analysis. Analysis of the resultant spatial and molecular data showed significant correlation between spatial and molecular heterogeneity at both transcriptomic and genomic level. This finding poses a notable challenge to conventional ITH studies, in which sampling locations are arbitrarily chosen without recording spatial details.

Several computational approaches, including genome-based (such as MATH [72], PyClone [73], and EXPANDS [74]) and transcriptome-based approaches (such as DEPTH [23] and tITH [75]), have been developed to estimate the degree of ITH, which are implemented based on single-region-derived data. Our results showed that estimated ITH varied significantly across different intra-tumor regions, indicating that single-region-based ITH estimation might be unreliable. A novel metric, normalized diversity score, was thus proposed to deliver a more accurate evaluation of patient-wise ITH. Compared with raw diversity score, normalized score is more likely to be related to tumor inherent properties rather than tumor size, and thus might have more biological relevance. According to this metric, HCC tumors were stratified into low-ITH and high-ITH classes. Consistent with previous reports, we observed that low-ITH tumors were associated with an immune-inflamed phenotype that was characterized by increased infiltration of TIL and higher inflammatory activity [76–78]. Interestingly, differential analysis of VIPER-inferred protein activity suggested that low-ITH tumors also exhibited higher PD-L1 activity than high-ITH tumors. Of note, PD-L1 inhibitor atezolizumab in combination with bevacizumab has already been approved for the treatment of patients with advanced HCC [79]. It thus could be speculated that this therapy is more likely to be effective in patients with low-ITH HCC, although further validation is required.

Currently, the degree of the overall immune ITH in HCC remains controversial. Besides, the comparisons of heterogeneity of different cell types within the tumor microenvironment have also not yet been reported. In this study, through the newly proposed metrics IHS, the heterogeneity of 22 CIBERSORT-estimated immune cell types was determined. Based on the IHS results, B cells seemed to be the most heterogenous cell type in HCC. The immune cell heterogeneity was also inspected in other four solid tumor types, and the results demonstrated that the phenomenon of high ITH of B cells was also present beyond HCC. Notably, B cells have been demonstrated to be closely related to the formation of tertiary lymphoid structures (TLSs), an ectopic lymphoid organ within tumors that engage in antitumor immune response [80]. Our findings suggested that B cells might not be an appropriate biomarker for identifying TLS due to the high heterogeneity in intra-tumor distribution. In addition, we also revealed an overall high immune heterogeneity in HCC via computational analyses combined with IHC validation. Contrary to a previous report, our results indicated that the evaluation of overall immune status of HCC based on single-region samples might not be reliable [36]. Furthermore, the ITH of protein activity of immune checkpoint genes was also investigated. Some low-ITH checkpoint genes, such as CD40 and CD47, might be promising therapeutic targets for the treatment of HCC.

Predictive models for risk stratification can help to develop individualized medicine and guide stratified treatment in clinics. For example, low-risk patients can be subjected to relatively conservative therapies to avoid overtreatment, whereas high-risk patients can be treated aggressively to ensure a favorable outcome. Although a plethora of predictive signatures for HCC have been developed over the last decade, none of them have been recommended for clinical use or commercialized as prognostic tests [33, 63–67]. These signatures were all susceptible to sampling bias due to the presence of ITH, since they were built on single-region-derived clinical cohorts and did not account for this issue during development. To overcome the hurdle of ITH in prognosis prediction, one feasible approach was to exclude high-ITH genes in advance when developing the model. Leveraging this approach, a previous study constructed a signature ORACLE for prognostication in lung cancer [27]. To the best of our knowledge, there are no similar signatures available in HCC to date. Thus, we developed LHRS, a predictive signature comprised of 18 low-ITH genes. Comprehensive validations confirmed that LHRS not only outperformed other published signatures in risk prediction but could also minimize the ITH bias.

This study has several limitations. First, the SLS strategy cannot discern the coordinates in the *z*-axis. Although some devices, such as neuronavigation system used in neurosurgery, can help to address this issue, these high-tech tools are not always available when needed and can be more cumbersome to implement compared with the SLS strategy. Second, because of the operational complexity and economic cost, implementing the SLS strategy in routine clinical practice is nearly impossible. Therefore, more convenient methods for accurately quantifying ITH are still warranted. Third, WES sequencing was performed on only partial samples. Despite numerous efforts to mitigate the effects of limited sequencing data, the findings in this study must be validated in future studies.

## Conclusions

In conclusion, this work discovered novel biological properties of low-ITH and high-ITH tumors and characterized the heterogeneity of immune features within tumor microenvironment, providing new insight into the complex phenotypes of HCC. A new LHRS signature was developed to overcome potential ITH bias in prognostication, which could have possible clinical applications.

## Supplementary Information

**Additional file 1.** Supplementary Figures 1-19.

**Additional file 2.** Supplementary Methods.

**Additional file 3:** **Table S1.** Clinical information of included patients. **Table S2.** Spatial coordinates of each sample. **Table S3.** Prediction results of CopyKAT. **Table S4.** ITH level of each patient. **Table S5.** Differentially expressed genes between high-ITH and low-ITH patients. **Table S6.** KEGG analysis of the differences between high-ITH and low-ITH patients. **Table S7.** IHS of each protein coding gene. **Table S8.** Included public datasets. **Table S9.** Summary of LHRS genes. **Table S10.** Summary of published prognostic signatures.

## Data Availability

Sequencing data generated in this study has been deposited at Zenodo through the https://doi.org/10.5281/zenodo.7336311 (https://zenodo.org/record/7336311#.Y3g5l-RBzZS) [81] and the National Omics Data Encyclopedia (NODE) under the accession code OEP002956 (http://www.biosino.org/node/project/detail/OEP002956) [82]. Key codes are available at https://github.com/YangJAT/LHRS [83]. Other public datasets have been listed in the Methods as well as Additional files.

## References

[1] Llovet JM, Kelley RK, Villanueva A, Singal AG, Pikarsky E, Roayaie S (2021). Hepatocellular carcinoma. Nat Rev Dis Primers.

[2] Yang C, Zhang H, Zhang L, Zhu AX, Bernards R, Qin W, et al. Evolving therapeutic landscape of advanced hepatocellular carcinoma. Nat Rev Gastroenterol Hepatol. 2022. 10.1038/s41575-022-00704-9 10.1038/s41575-022-00704-9 36369487

[3] Akhoundi M, Mohammadi M, Sahraei SS, Sheykhhasan M, Fayazi N (2021). CAR T cell therapy as a promising approach in cancer immunotherapy: challenges and opportunities. Cell Oncol (Dordr).

[4] Yang C, Guo Y, Qian R, Huang Y, Zhang L, Wang J (2021). Mapping the landscape of synthetic lethal interactions in liver cancer. Theranostics.

[5] Yang C, Zhang H, Chen M, Wang S, Qian R, Zhang L (2022). A survey of optimal strategy for signature-based drug repositioning and an application to liver cancer. Elife..

[6] Marusyk A, Janiszewska M, Polyak K (2020). Intratumor heterogeneity: the Rosetta Stone of Therapy Resistance. Cancer Cell.

[7] Craig AJ, von Felden J, Garcia-Lezana T, Sarcognato S, Villanueva A (2020). Tumour evolution in hepatocellular carcinoma. Nat Rev Gastroenterol Hepatol.

[8] Rebouissou S, Nault JC (2020). Advances in molecular classification and precision oncology in hepatocellular carcinoma. J Hepatol.

[9] Torrecilla S, Sia D, Harrington AN, Zhang Z, Cabellos L, Cornella H (2017). Trunk mutational events present minimal intra- and inter-tumoral heterogeneity in hepatocellular carcinoma. J Hepatol.

[10] Dong LQ, Peng LH, Ma LJ, Liu DB, Zhang S, Luo SZ (2020). Heterogeneous immunogenomic features and distinct escape mechanisms in multifocal hepatocellular carcinoma. J Hepatol.

[11] Friemel J, Rechsteiner M, Frick L, Böhm F, Struckmann K, Egger M (2015). Intratumor heterogeneity in hepatocellular carcinoma. Clin Cancer Res.

[12] Ding X, He M, Chan AWH, Song QX, Sze SC, Chen H (2019). Genomic and epigenomic features of primary and recurrent hepatocellular carcinomas. Gastroenterol.

[13] Zhang Q, Lou Y, Yang J, Wang J, Feng J, Zhao Y (2019). Integrated multiomic analysis reveals comprehensive tumour heterogeneity and novel immunophenotypic classification in hepatocellular carcinomas. Gut.

[14] Losic B, Craig AJ, Villacorta-Martin C, Martins-Filho SN, Akers N, Chen X (2020). Intratumoral heterogeneity and clonal evolution in liver cancer. Nat Commun.

[15] Lin DC, Mayakonda A, Dinh HQ, Huang P, Lin L, Liu X (2017). Genomic and epigenomic heterogeneity of hepatocellular carcinoma. Cancer Res.

[16] Buczak K, Ori A, Kirkpatrick JM, Holzer K, Dauch D, Roessler S (2018). Spatial tissue proteomics quantifies inter- and intratumor heterogeneity in hepatocellular carcinoma (HCC). Mol Cell Proteomics.

[17] Zhang Q, Lou Y, Bai XL, Liang TB (2020). Intratumoral heterogeneity of hepatocellular carcinoma: from single-cell to population-based studies. World J Gastroenterol.

[18] Gao Q, Wang ZC, Duan M, Lin YH, Zhou XY, Worthley DL (2017). Cell culture system for analysis of genetic heterogeneity within hepatocellular carcinomas and response to pharmacologic agents. Gastroenterol.

[19] Li L, Knutsdottir H, Hui K, Weiss MJ, He J, Philosophe B, et al. Human primary liver cancer organoids reveal intratumor and interpatient drug response heterogeneity. JCI Insight. 2019;4. 10.1172/jci.insight.121490 10.1172/jci.insight.121490 PMC6413833 30674722

[20] Huang A, Zhao X, Yang XR, Li FQ, Zhou XL, Wu K (2017). Circumventing intratumoral heterogeneity to identify potential therapeutic targets in hepatocellular carcinoma. J Hepatol.

[21] Nguyen PHD, Ma S, Phua CZJ, Kaya NA, Lai HLH, Lim CJ (2021). Intratumoural immune heterogeneity as a hallmark of tumour evolution and progression in hepatocellular carcinoma. Nat Commun.

[22] Ma L, Hernandez MO, Zhao Y, Mehta M, Tran B, Kelly M (2019). Tumor cell biodiversity drives microenvironmental reprogramming in liver cancer. Cancer Cell.

[23] Li M, Zhang Z, Li L, Wang X (2020). An algorithm to quantify intratumor heterogeneity based on alterations of gene expression profiles. Commun Biol.

[24] Bachtiary B, Boutros PC, Pintilie M, Shi W, Bastianutto C, Li JH (2006). Gene expression profiling in cervical cancer: an exploration of intratumor heterogeneity. Clin Cancer Res.

[25] Yan W, Shih J, Rodriguez-Canales J, Tangrea MA, Player A, Diao L (2013). Three-dimensional mRNA measurements reveal minimal regional heterogeneity in esophageal squamous cell carcinoma. Am J Pathol.

[26] Dunne PD, Alderdice M, O'Reilly PG, Roddy AC, McCorry AMB, Richman S (2017). Cancer-cell intrinsic gene expression signatures overcome intratumoural heterogeneity bias in colorectal cancer patient classification. Nat Commun.

[27] Biswas D, Birkbak NJ, Rosenthal R, Hiley CT, Lim EL, Papp K (2019). A clonal expression biomarker associates with lung cancer mortality. Nat Med.

[28] Gao Q, Zhu H, Dong L, Shi W, Chen R, Song Z (2019). Integrated proteogenomic characterization of HBV-related hepatocellular carcinoma. Cell.

[29] Schulze K, Imbeaud S, Letouzé E, Alexandrov LB, Calderaro J, Rebouissou S (2015). Exome sequencing of hepatocellular carcinomas identifies new mutational signatures and potential therapeutic targets. Nat Genet.

[30] Fujimoto A, Furuta M, Totoki Y, Tsunoda T, Kato M, Shiraishi Y (2016). Whole-genome mutational landscape and characterization of noncoding and structural mutations in liver cancer. Nat Genet.

[31] Ally A, Balasundaram M, Carlsen R, Chuah E, Clarke A, Dhalla N (2017). Comprehensive and integrative genomic characterization of hepatocellular carcinoma. Cell.

[32] Roessler S, Long EL, Budhu A, Chen Y, Zhao X, Ji J (2012). Integrative genomic identification of genes on 8p associated with hepatocellular carcinoma progression and patient survival. Gastroenterol.

[33] Villa E, Critelli R, Lei B, Marzocchi G, Cammà C, Giannelli G (2016). Neoangiogenesis-related genes are hallmarks of fast-growing hepatocellular carcinomas and worst survival Results from a prospective study. Gut.

[34] Li B, Dewey CN (2011). RSEM: accurate transcript quantification from RNA-Seq data with or without a reference genome. BMC Bioinform.

[35] Liu J, Lichtenberg T, Hoadley KA, Poisson LM, Lazar AJ, Cherniack AD (2018). An integrated TCGA pan-cancer clinical data resource to drive high-quality survival outcome analytics. Cell.

[36] Shen YC, Hsu CL, Jeng YM, Ho MC, Ho CM, Yeh CP (2020). Reliability of a single-region sample to evaluate tumor immune microenvironment in hepatocellular carcinoma. J Hepatol.

[37] Villanueva A, Hoshida Y, Battiston C, Tovar V, Sia D, Alsinet C (2011). Combining clinical, pathology, and gene expression data to predict recurrence of hepatocellular carcinoma. Gastroenterol.

[38] Shi L, Zhang Y, Feng L, Wang L, Rong W, Wu F (2017). Multi-omics study revealing the complexity and spatial heterogeneity of tumor-infiltrating lymphocytes in primary liver carcinoma. Oncotarget.

[39] Kim D, Paggi JM, Park C, Bennett C, Salzberg SL (2019). Graph-based genome alignment and genotyping with HISAT2 and HISAT-genotype. Nat Biotechnol.

[40] Liao Y, Smyth GK, Shi W (2013). The Subread aligner: fast, accurate and scalable read mapping by seed-and-vote. Nucleic Acids Res.

[41] Barry WT, Kernagis DN, Dressman HK, Griffis RJ, Hunter JD, Olson JA (2010). Intratumor heterogeneity and precision of microarray-based predictors of breast cancer biology and clinical outcome. J Clin Oncol.

[42] Quek K, Li J, Estecio M, Zhang J, Fujimoto J, Roarty E (2017). DNA methylation intratumor heterogeneity in localized lung adenocarcinomas. Oncotarget.

[43] Morrissy AS, Cavalli FMG, Remke M, Ramaswamy V, Shih DJH, Holgado BL (2017). Spatial heterogeneity in medulloblastoma. Nat Genet.

[44] Stuart T, Butler A, Hoffman P, Hafemeister C, Papalexi E, Mauck WM (2019). Comprehensive integration of single-cell data. Cell.

[45] Gao R, Bai S, Henderson YC, Lin Y, Schalck A, Yan Y (2021). Delineating copy number and clonal substructure in human tumors from single-cell transcriptomes. Nat Biotechnol.

[46] Fox J, Carvalho MS (2012). The RcmdrPlugin.survival Package: extending the R Commander interface to survival analysis. J Stat Softw..

[47] Blanche P, Dartigues JF, Jacqmin-Gadda H (2013). Estimating and comparing time-dependent areas under receiver operating characteristic curves for censored event times with competing risks. Stat Med.

[48] Ho DW, Tsui YM, Chan LK, Sze KM, Zhang X, Cheu JW (2021). Single-cell RNA sequencing shows the immunosuppressive landscape and tumor heterogeneity of HBV-associated hepatocellular carcinoma. Nat Commun.

[49] Sun Y, Wu L, Zhong Y, Zhou K, Hou Y, Wang Z (2021). Single-cell landscape of the ecosystem in early-relapse hepatocellular carcinoma. Cell.

[50] Sharma A, Merritt E, Hu X, Cruz A, Jiang C, Sarkodie H (2019). Non-genetic intra-tumor heterogeneity is a major predictor of phenotypic heterogeneity and ongoing evolutionary dynamics in lung tumors. Cell Rep.

[51] Iacobuzio-Donahue CA, Litchfield K, Swanton C (2020). Intratumor heterogeneity reflects clinical disease course. Nat Cancer.

[52] Gerlinger M, Rowan AJ, Horswell S, Math M, Larkin J, Endesfelder D (2012). Intratumor heterogeneity and branched evolution revealed by multiregion sequencing. N Engl J Med.

[53] Gerlinger M, Horswell S, Larkin J, Rowan AJ, Salm MP, Varela I (2014). Genomic architecture and evolution of clear cell renal cell carcinomas defined by multiregion sequencing. Nat Genet.

[54] Jamal-Hanjani M, Wilson GA, McGranahan N, Birkbak NJ, Watkins TBK, Veeriah S (2017). Tracking the evolution of non-small-cell lung cancer. N Engl J Med.

[55] Newman AM, Liu CL, Green MR, Gentles AJ, Feng W, Xu Y (2015). Robust enumeration of cell subsets from tissue expression profiles. Nat Methods.

[56] Paijens ST, Vledder A, de Bruyn M, Nijman HW (2021). Tumor-infiltrating lymphocytes in the immunotherapy era. Cell Mol Immunol.

[57] Alvarez MJ, Shen Y, Giorgi FM, Lachmann A, Ding BB, Ye BH (2016). Functional characterization of somatic mutations in cancer using network-based inference of protein activity. Nat Genet.

[58] Obradovic A, Vlahos L, Laise P, Worley J, Tan X, Wang A (2021). PISCES: A pipeline for the systematic, protein activity-based analysis of single cell RNA sequencing data. bioRxiv.

[59] Kolde R, Laur S, Adler P, Vilo J (2012). Robust rank aggregation for gene list integration and meta-analysis. Bioinform.

[60] Yoshihara K, Shahmoradgoli M, Martínez E, Vegesna R, Kim H, Torres-Garcia W (2013). Inferring tumour purity and stromal and immune cell admixture from expression data. Nat Commun.

[61] Hayat SMG, Bianconi V, Pirro M, Jaafari MR, Hatamipour M, Sahebkar A (2020). CD47: role in the immune system and application to cancer therapy. Cell Oncol (Dordr).

[62] Vonderheide RH (2020). CD40 agonist antibodies in cancer immunotherapy. Annu Rev Med.

[63] van Malenstein H, Gevaert O, Libbrecht L, Daemen A, Allemeersch J, Nevens F (2010). A seven-gene set associated with chronic hypoxia of prognostic importance in hepatocellular carcinoma. Clin Cancer Res.

[64] Nault JC, De Reyniès A, Villanueva A, Calderaro J, Rebouissou S, Couchy G (2013). A hepatocellular carcinoma 5-gene score associated with survival of patients after liver resection. Gastroenterology.

[65] Fang Q, Chen H (2020). Development of a novel autophagy-related prognostic signature and nomogram for hepatocellular carcinoma. Front Oncol.

[66] Fang Q, Chen H (2020). The significance of m6A RNA methylation regulators in predicting the prognosis and clinical course of HBV-related hepatocellular carcinoma. Mol Med.

[67] Pan Q, Qin F, Yuan H, He B, Yang N, Zhang Y (2021). Normal tissue adjacent to tumor expression profile analysis developed and validated a prognostic model based on Hippo-related genes in hepatocellular carcinoma. Cancer Med.

[68] Kudo M, Izumi N, Ichida T, Ku Y, Kokudo N, Sakamoto M (2016). Report of the 19th follow-up survey of primary liver cancer in Japan. Hepatol Res.

[69] Zhai W, Lai H, Kaya NA, Chen J, Yang H, Lu B (2022). Dynamic phenotypic heterogeneity and the evolution of multiple RNA subtypes in hepatocellular carcinoma: the PLANET study. Natl Sci Rev.

[70] Bassiouni R, Gibbs LD, Craig DW, Carpten JD, McEachron TA (2021). Applicability of spatial transcriptional profiling to cancer research. Mol Cell.

[71] Mitra A, Andrews MC, Roh W, De Macedo MP, Hudgens CW, Carapeto F (2020). Spatially resolved analyses link genomic and immune diversity and reveal unfavorable neutrophil activation in melanoma. Nat Commun.

[72] Mroz EA, Rocco JW (2013). MATH, a novel measure of intratumor genetic heterogeneity, is high in poor-outcome classes of head and neck squamous cell carcinoma. Oral Oncol.

[73] Roth A, Khattra J, Yap D, Wan A, Laks E, Biele J (2014). PyClone: statistical inference of clonal population structure in cancer. Nat Methods.

[74] Andor N, Harness JV, Müller S, Mewes HW, Petritsch C (2014). EXPANDS: expanding ploidy and allele frequency on nested subpopulations. Bioinformatics.

[75] Park Y, Lim S, Nam JW, Kim S (2016). Measuring intratumor heterogeneity by network entropy using RNA-seq data. Sci Rep.

[76] Wolf Y, Samuels Y (2022). Intratumor heterogeneity and antitumor immunity shape one another bidirectionally. Clin Cancer Res.

[77] Ran X, Xiao J, Zhang Y, Teng H, Cheng F, Chen H (2020). Low intratumor heterogeneity correlates with increased response to PD-1 blockade in renal cell carcinoma. Ther Adv Med Oncol.

[78] Lin Z, Meng X, Wen J, Corral JM, Andreev D, Kachler K (2020). Intratumor heterogeneity correlates with reduced immune activity and worse survival in melanoma patients. Front Oncol.

[79] Cheng AL, Qin S, Ikeda M, Galle PR, Ducreux M, Kim TY (2022). Updated efficacy and safety data from IMbrave150: atezolizumab plus bevacizumab vs. sorafenib for unresectable hepatocellular carcinoma. J Hepatol.

[80] Schumacher TN, Thommen DS (2022). Tertiary lymphoid structures in cancer. Sci.

[81] Yang C, Wang C, Hang H. Multi-region sequencing with spatial information enables accurate heterogeneity estimation and risk stratification in liver cancer. 10.5281/zenodo.7336311, Zenodo. 2022. https://zenodo.org/record/7336311#.Y3g5l-RBzZS. Accessed 19 Nov 2022. 10.1186/s13073-022-01143-6 PMC9758830 36527145

[82] Yang C, Wang C, Hang H. Multi-region sequencing with spatial information enables accurate heterogeneity estimation and risk stratification in liver cancer. OEP002956, National Omics Data Encyclopedia. 2022. http://www.biosino.org/node/project/detail/OEP002956. Accessed 3 Jan 2022. 10.1186/s13073-022-01143-6 PMC9758830 36527145

[83] Yang C, Wang C, Hang H. Github. 2022. https://github.com/YangJAT/LHRS. Accessed 27 Nov 2022.

