# Supplementary material for: Multi-region sequencing with spatial information enables accurate heterogeneity estimation and risk stratification in liver cancer
Source: Genome Med. 2022 Dec 16;14:142. doi: 10.1186/s13073-022-01143-6 (PMC9758830; doi:10.1186/s13073-022-01143-6)
Supplement: Supplementary file 1 — Additional file 1. Supplementary Figures 1-19. [file 13073_2022_1143_MOESM1_ESM.pdf]

Supplementary Figures

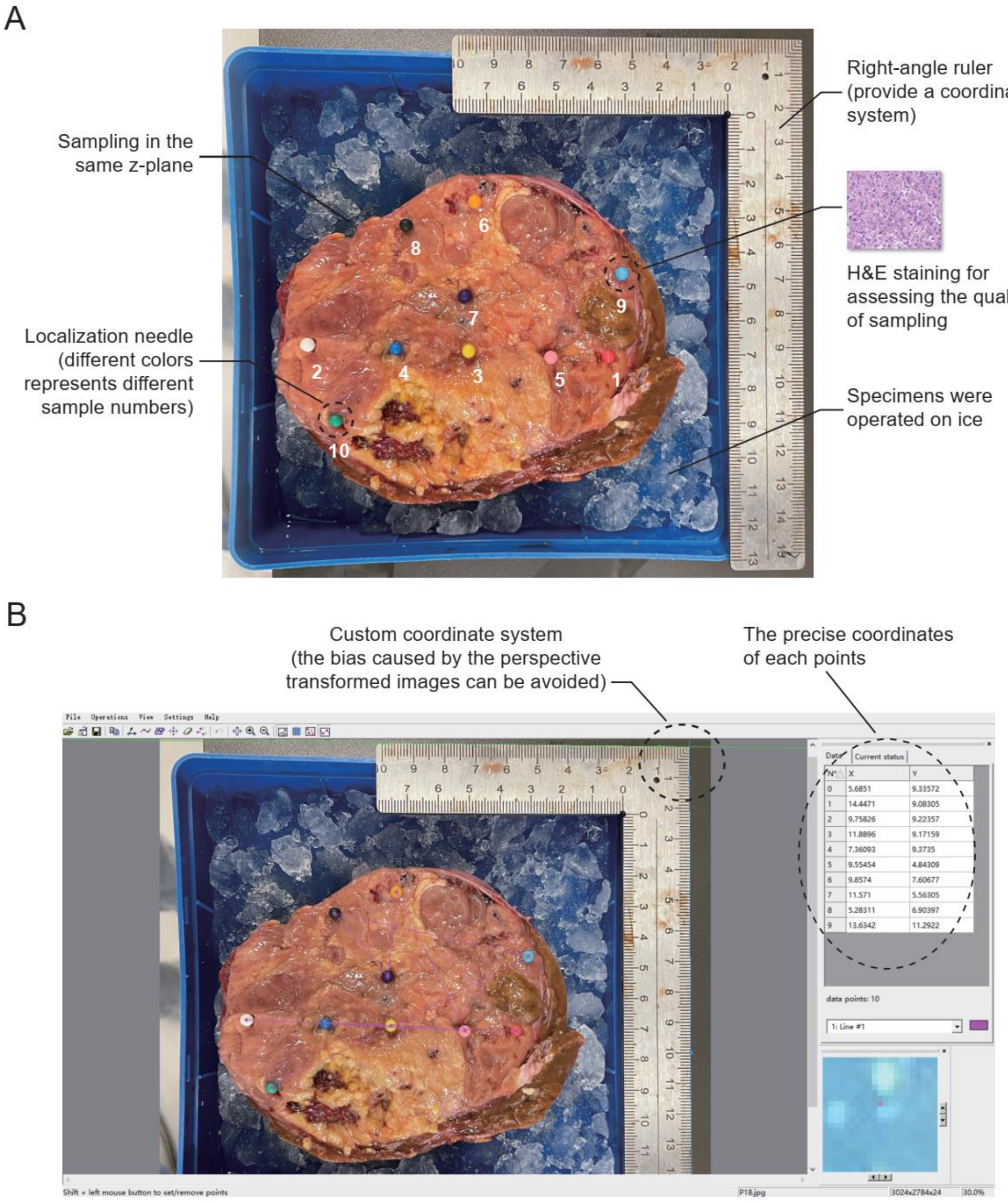

Figure S1. Schematic representation of the spatial localization sampling (SLS) strategy. (A) An example of clinical sample collection. All the sampling sites were marked using localization needle. (B) An example of spatial coordinates acquisition. GetData Graph Digitizer (version 2.26) was utilized to reconstruct the coordinate system and extract the coordinates.

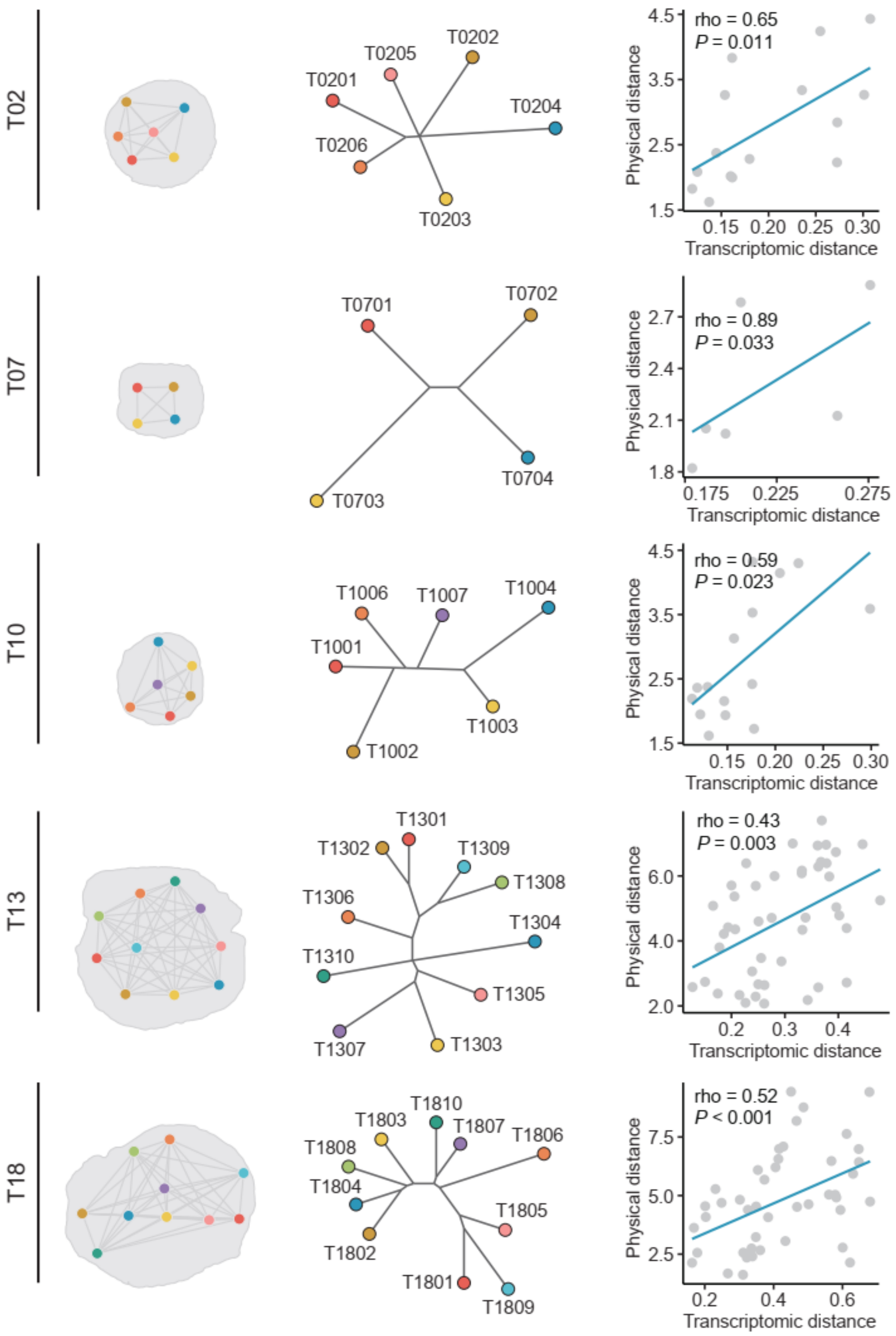

Figure S2. Individual patient-level correlation between transcriptomic and physical distance. Only patients with statistically significant results were presented. The left part of the figure shows the spatial distribution of sampling sites. The middle part shows the phylogenetic trees constructed on gene expression data. The branch length correlated with the transcriptomic similarities. The right part shows the statistical analysis of Spearman correlation between transcriptomic distance and physical distance.

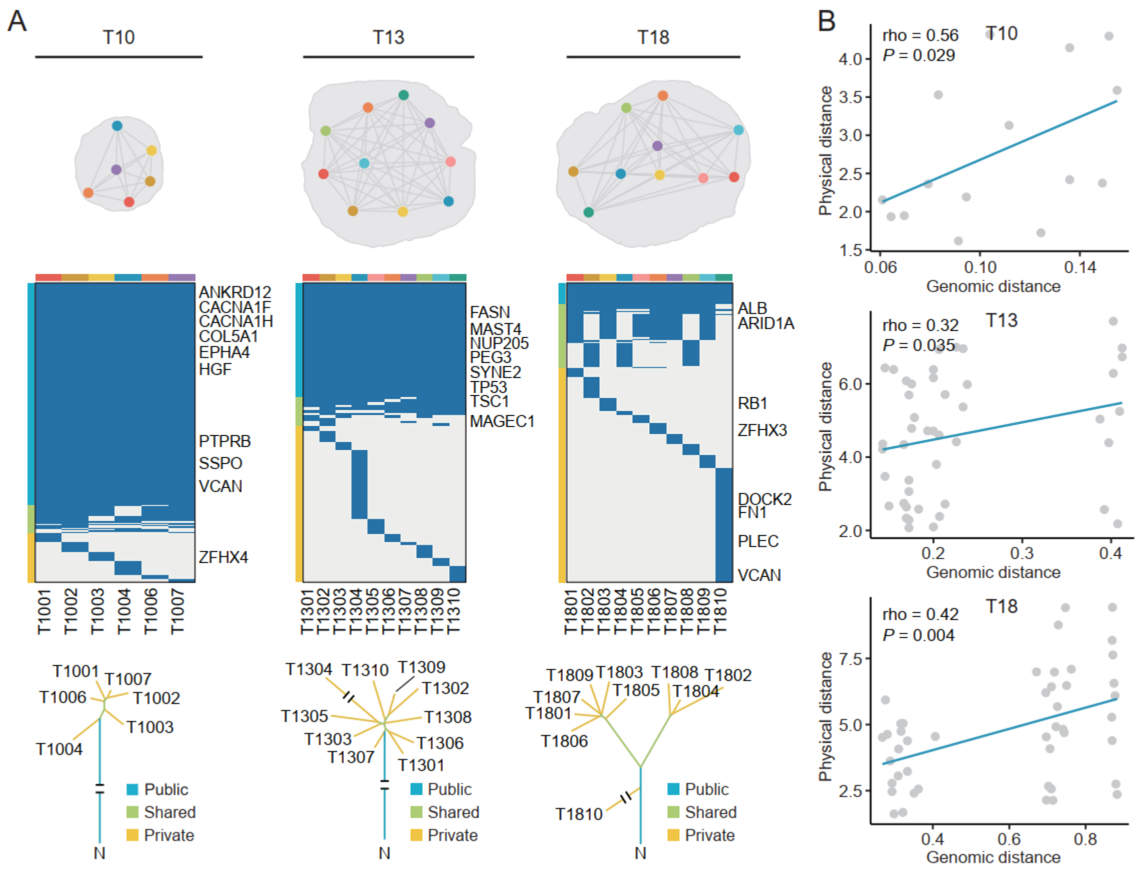

Figure S3. Individual patient-level correlation between genomic and physical distance. (A) Phylogenetic trees of three HCCs constructed on multi-region whole-exome sequencing (WES). The upper part of the figure shows the spatial distribution of sampling sites. The middle part shows the heatmaps that indicate the presence (blue) or absence (grey) of a mutation in each tumor region. The lower part shows the phylogenetic trees constructed on WES-based mutation data. The branch lengths are proportional to the number of mutations. (B) Statistical analysis of Spearman correlation between genomic distance and physical distance.

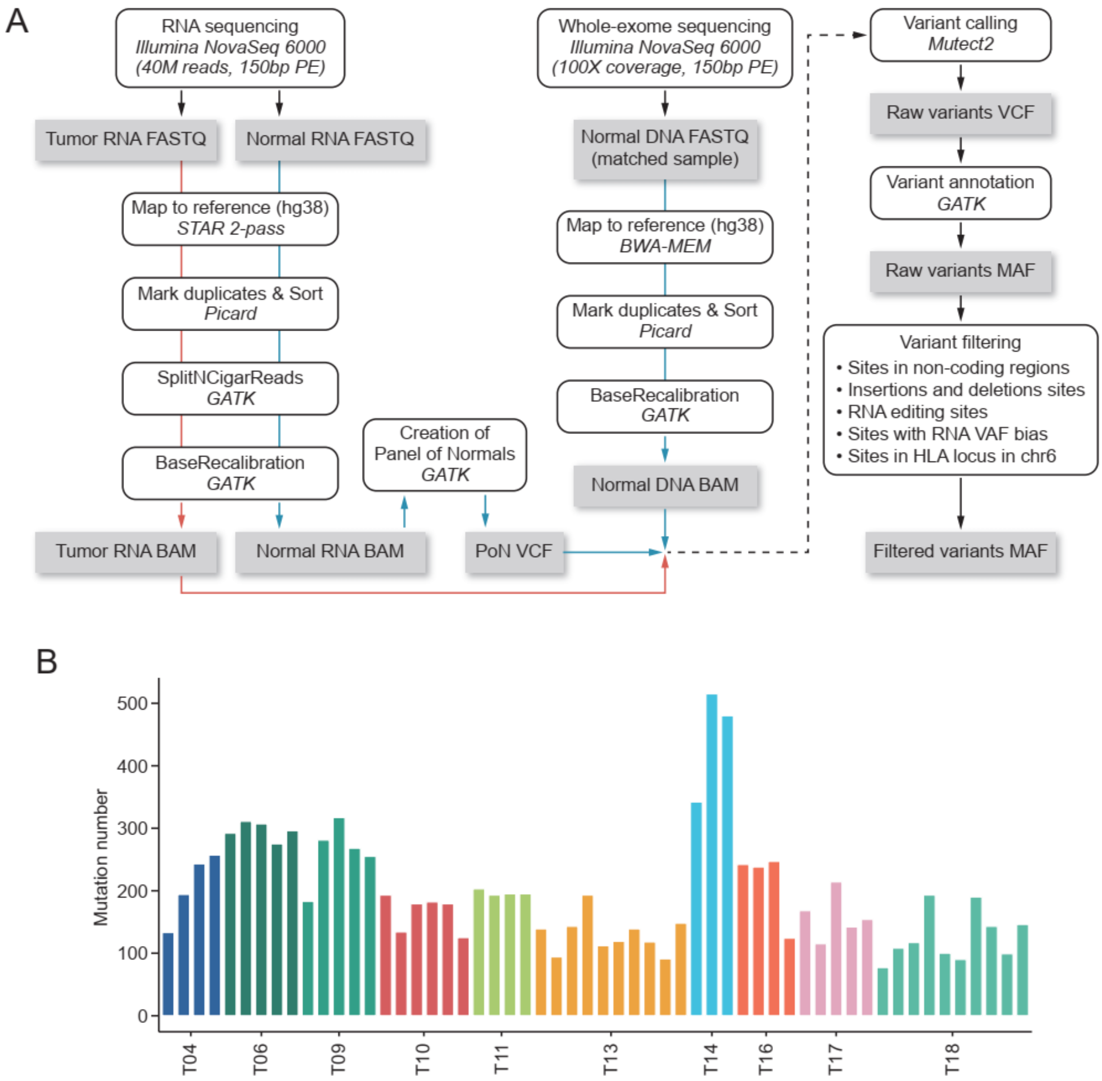

Figure S4. RNA sequencing data-based somatic mutation calling. (A) Flow chart of the calling procedure of somatic mutation based on tumor samples (RNA data) and matched normal samples (WES data). (B) The distribution of RNA-based mutation numbers across different tumors.

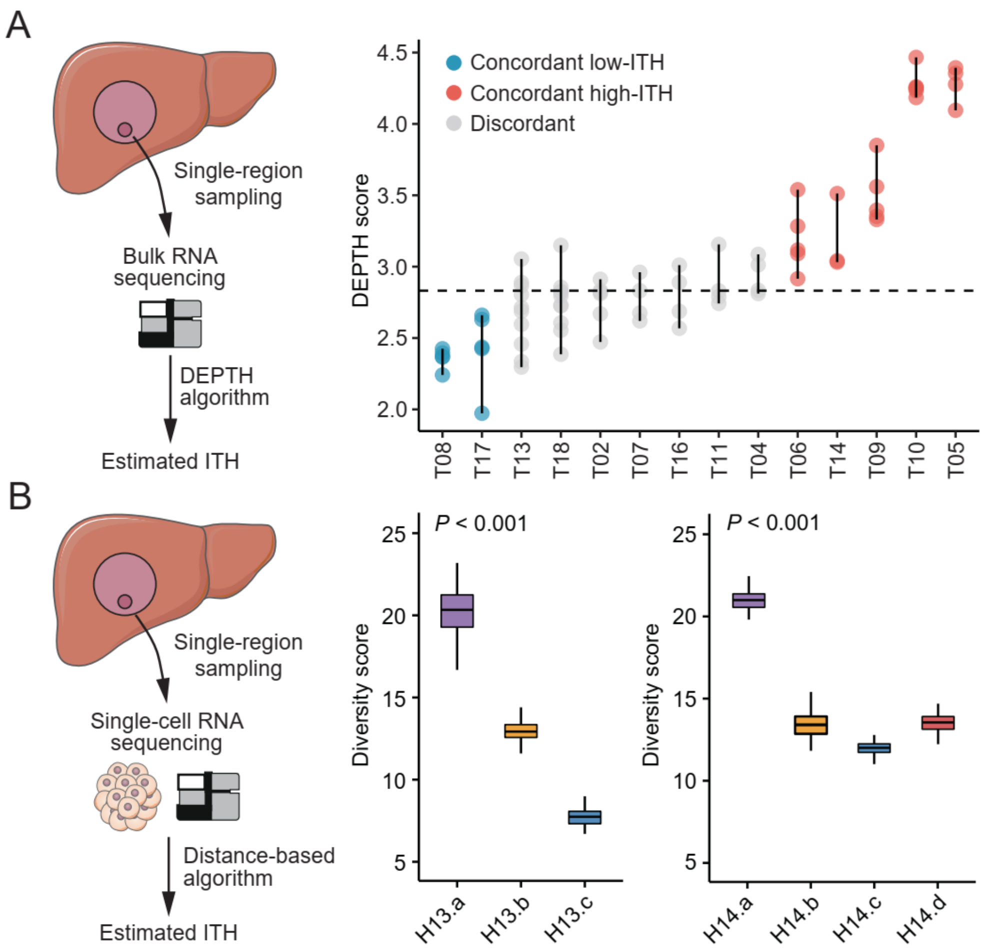

Figure S5. Assessment of previous approaches for measuring transcriptome-level intratumor heterogeneity (ITH). (A) An illustration of single-region bulk RNA sequencing-based ITH measurement. A recently proposed algorithm, deviating gene expression profiling tumor heterogeneity (DEPTH), was utilized to calculate the ITH score of tumors derived from different regions [1]. (B) An illustration of single-region single-cell RNA sequencing-based ITH measurement. A distance-based algorithm based on single-cell RNA data was utilized to determine the ITH score of tumors derived from different regions [2].

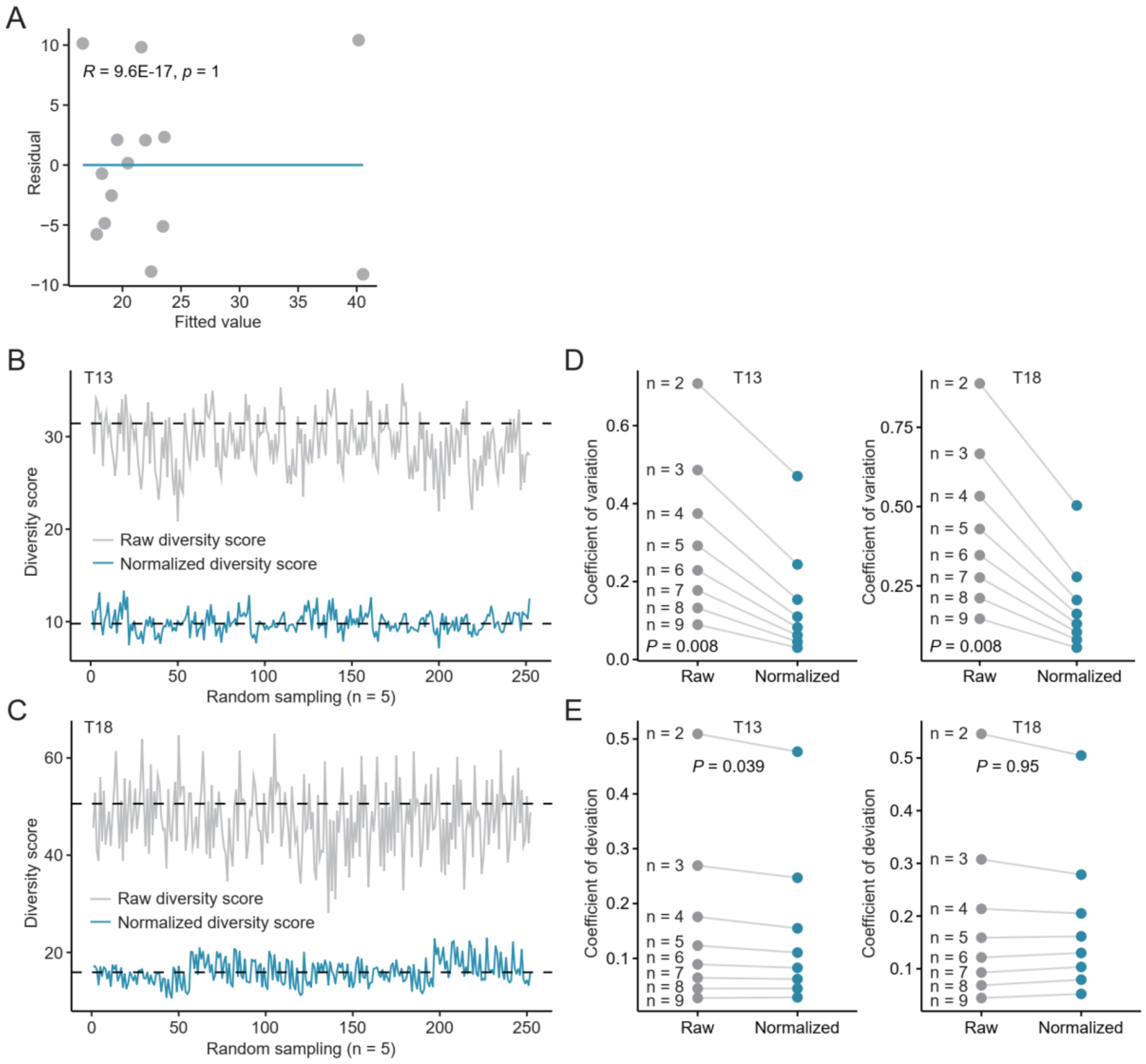

Figure S6. Calculation of normalized diversity scores. (A) Residuals versus fits plot showing the pattern of relationship. If the residuals and the fitted values are uncorrelated, then the relationship is linear. (B and C) Representative examples show the distribution of both raw and normalized diversity scores in the condition of setting the number of random samplings to 5 on tumor T13 (B) and T18 (C). For a given number (n = 5 in this case) of tumor samples, we numerated all combinations of n tumor samples to derive the distribution of diversity scores. The upper and lower dotted lines indicated the 'gold standard' (calculated on all the 10 tumors from different regions) of raw and normalized diversity scores, respectively. (D) Comparison of CV values across different sampling numbers between raw and normalized diversity scores. (E) Comparison of CD values across different sampling numbers between raw and normalized diversity scores.

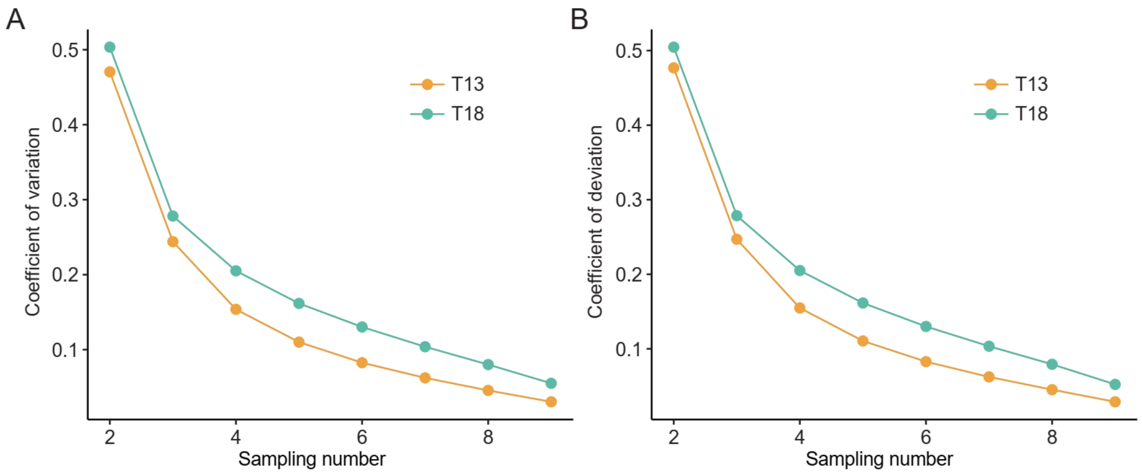

Figure S7. Investigating an appropriate sampling number for calculating normalized diversity scores. Coefficient of variation (A) and coefficient of deviation (B) of normalized diversity scores calculated on tumor T13 and T18 by sampling 2 to 9 biopsies.

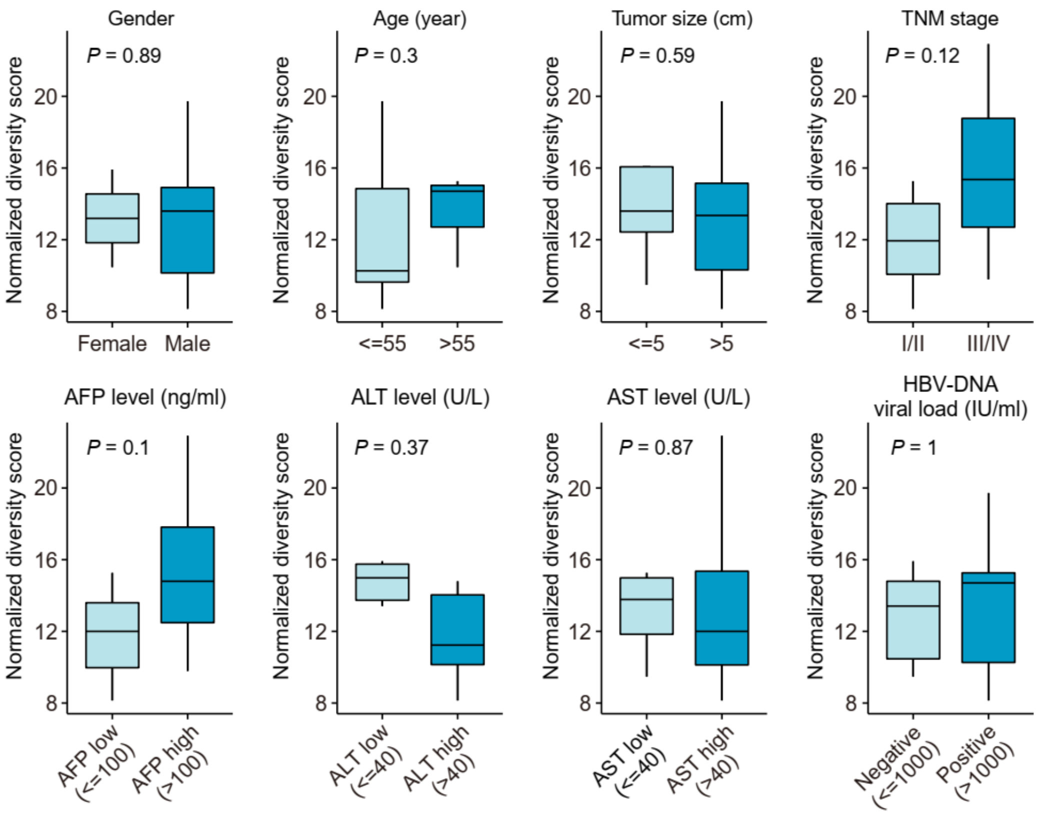

Figure S8. Relationship between intratumor heterogeneity and different clinical features. Eight clinical features, including gender, age, tumor size, TNM stage, AFP level, ALT level, AST level and HBV-DNA, were included for investigation. Non-dichotomous variables were transformed into dichotomous variables based on clinical judgment. Statistical significance of difference was determined using Wilcoxon rank-sum test.

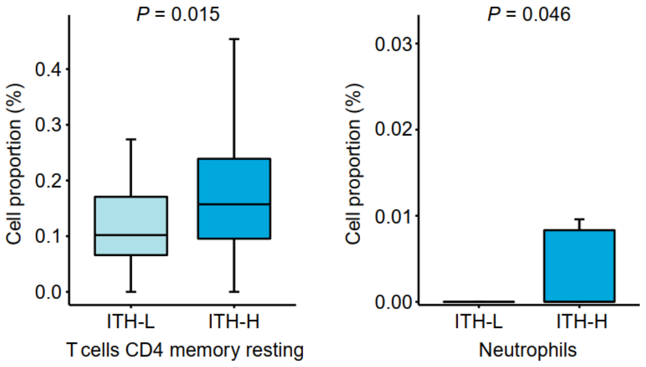

Figure S9. Differential infiltrated cell types between low-ITH and high-ITH tumors. Statistical significance of difference was determined using Wilcoxon rank-sum test.

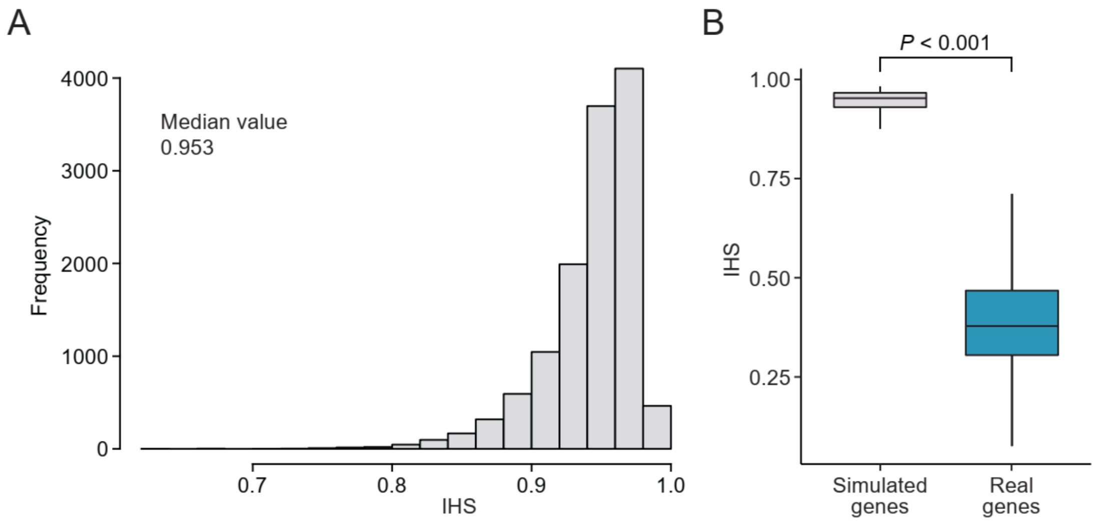

Figure S10. Investigation of IHS calculated on simulated expression values. (A) The distribution of IHS of simulated expression values. The simulated data distribution was fitted with the negative binomial distribution. (B) Comparison of IHS between simulated and real genes. Statistical significance of difference was determined using Wilcoxon rank-sum test.

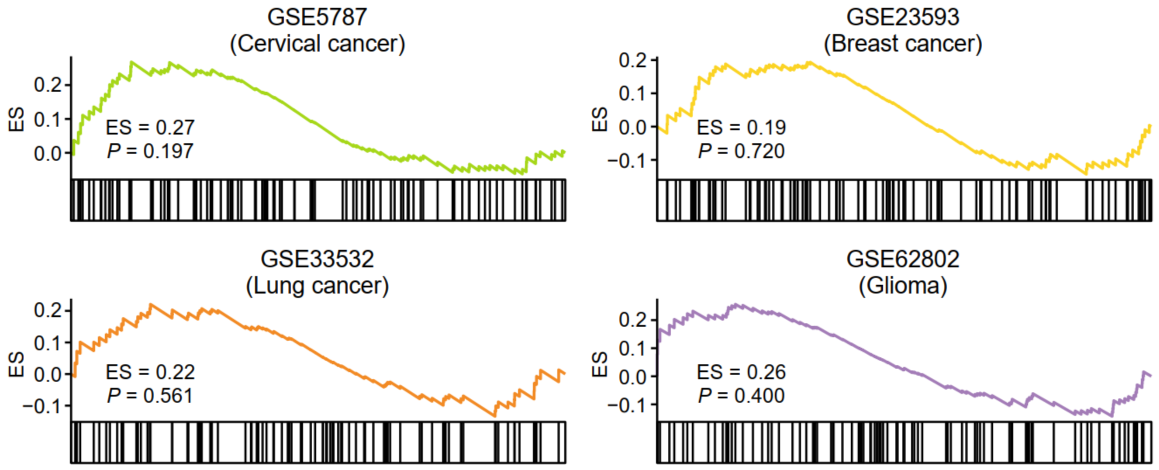

Figure S11. Enrichment results of top 100 genes with lowest IHS of our cohort against ranked IHS results from other four solid tumors, including cervical cancer (GSE5787), breast cancer (GSE23593), lung cancer (GSE33532) and high-grade glioma (GSE62802).

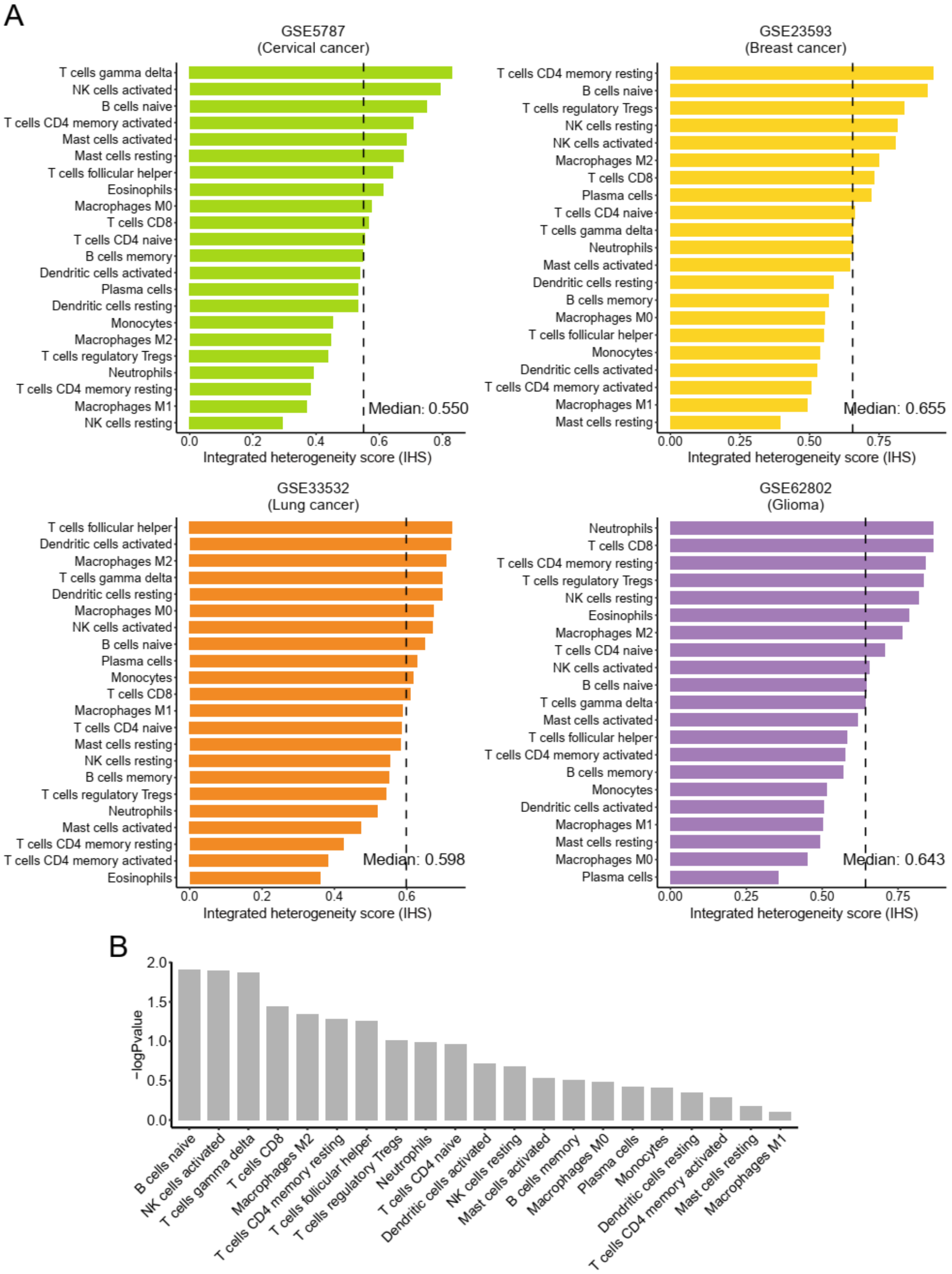

Figure S12. Immune cell heterogeneity in tumors beyond HCC. (A) IHS of 22 CIBERSORT-estimated immune cell types in four solid tumors, including cervical cancer (GSE5787), breast cancer (GSE23593), lung cancer (GSE33532) and high-grade glioma (GSE62802). (B) Integrated result of four solid tumors based on Robust Rank Aggregation (RRA) method. A lower p value indicates an overall higher IHS value (thus higher heterogeneity).

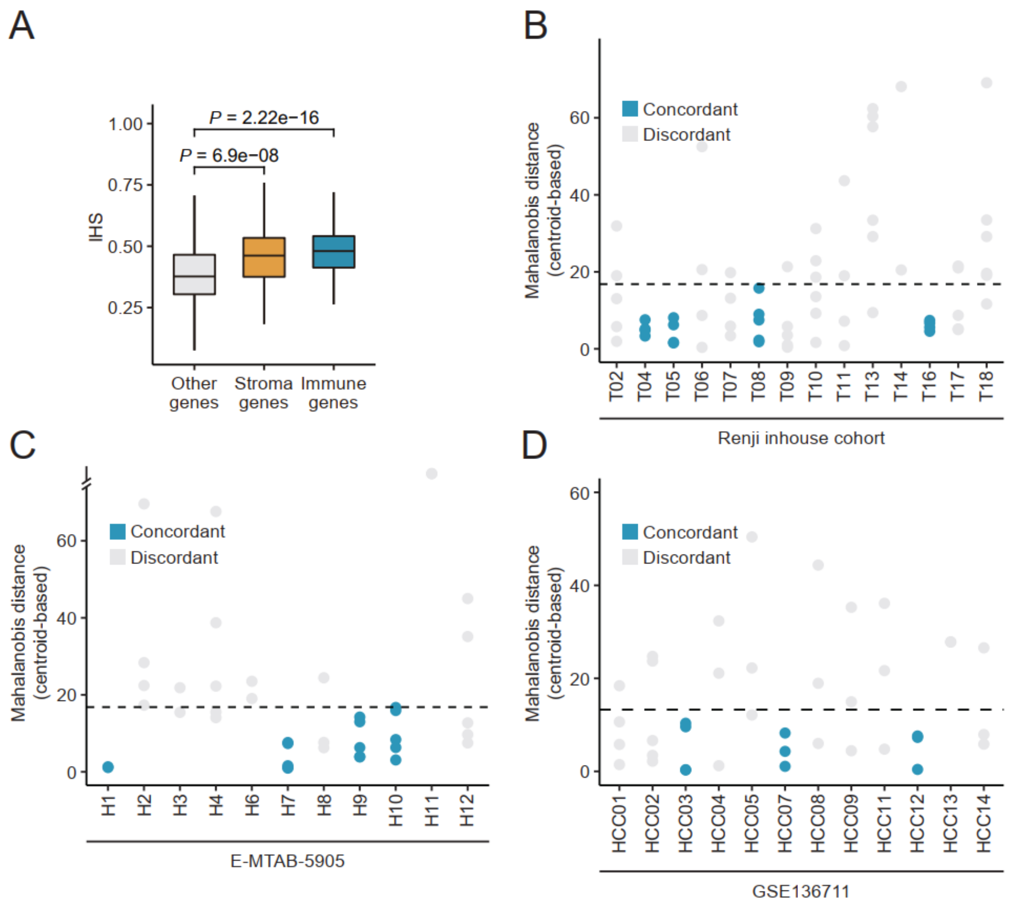

Figure S13. Quantitative assessment of immune heterogeneity in HCC (A) Comparison of IHS between tumor microenvironment-associated genes (including immune and stroma genes) and other genes. Statistical significance of difference was determined using Wilcoxon rank-sum test. (B-D) Similarities of immune-related expression profiles of different intra-tumor regions. Centroid-based Mahalanobis distance was utilized to measure the similarities between different regions. The y-axis values represent the distances between each point and the given center point. The dotted line denotes the threshold for determining whether the immune profiles of different regions within the same tumor are concordant. The threshold was calculated based on the Chi-Square value with 5 degrees of freedom (equivalent to the number of PCs generated by PCA) and 0.99 probability.

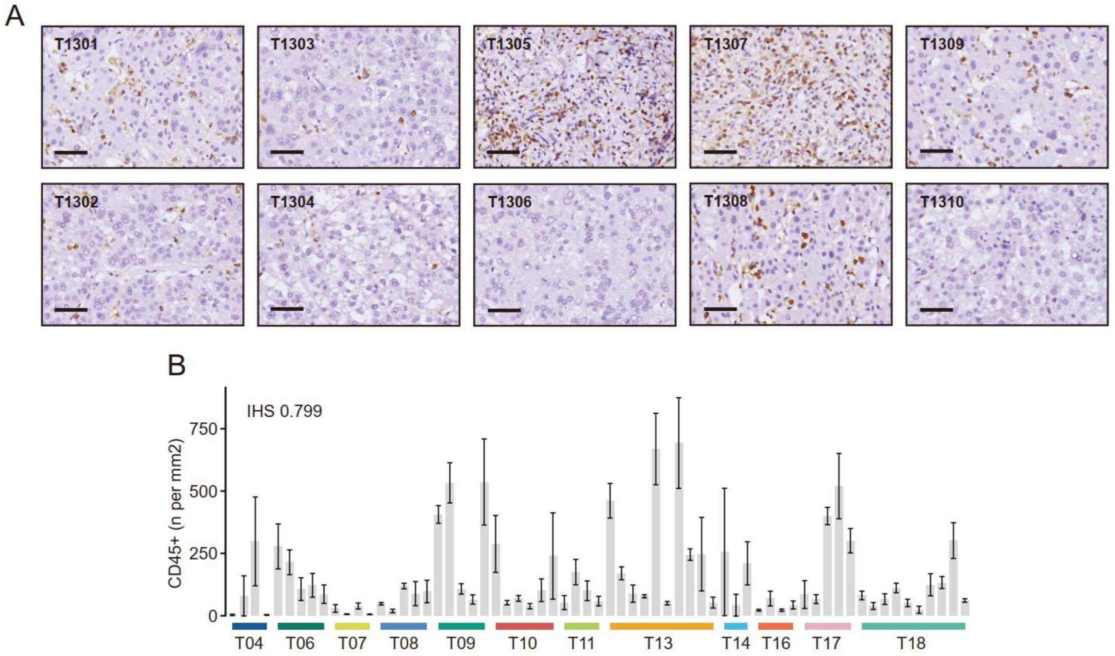

Figure S14. Validation of immune heterogeneity in HCC. (A) Representative images from patient T13 of immunohistochemistry (IHC) staining for CD45 (scale bars: 50 µm). (B) IHS of the CD45+ cell infiltration.

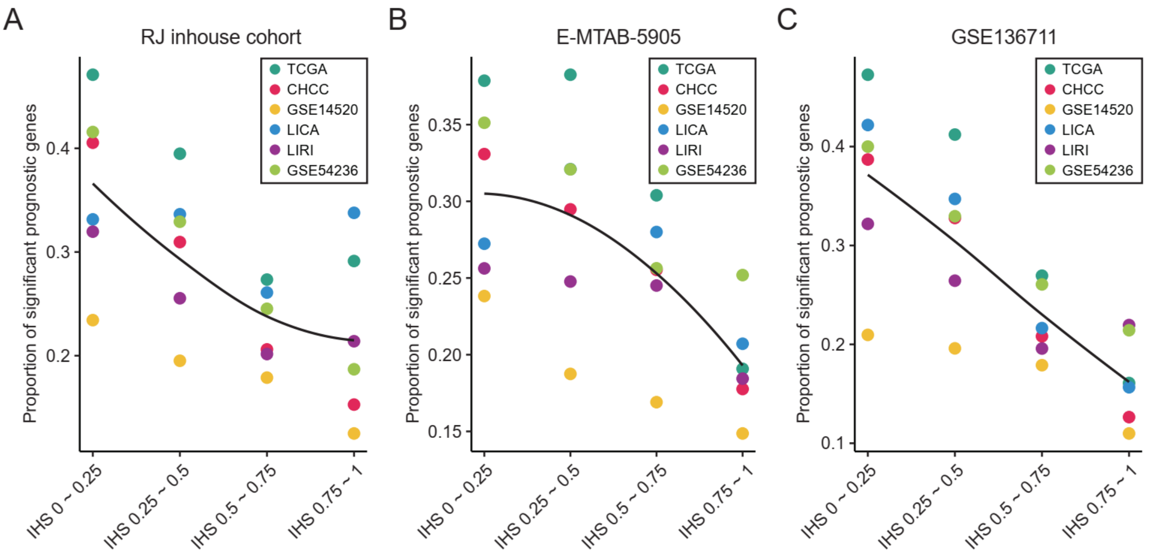

Figure S15. Association between IHS and prognostic significance. Three different multi-region sequencing cohorts, including RJ inhouse cohort (A), E-MTAB-5905 (B) and GSE136711 (C), were used to calculate the IHS of each gene. Genes were divided into four groups according to IHS. Six large-scale HCC cohorts with available survival data were utilized to identify significantly prognostic genes. The scatter plots show the proportion of significant genes in each gene group in each HCC cohort. The solid lines indicate the Loess regression fit.

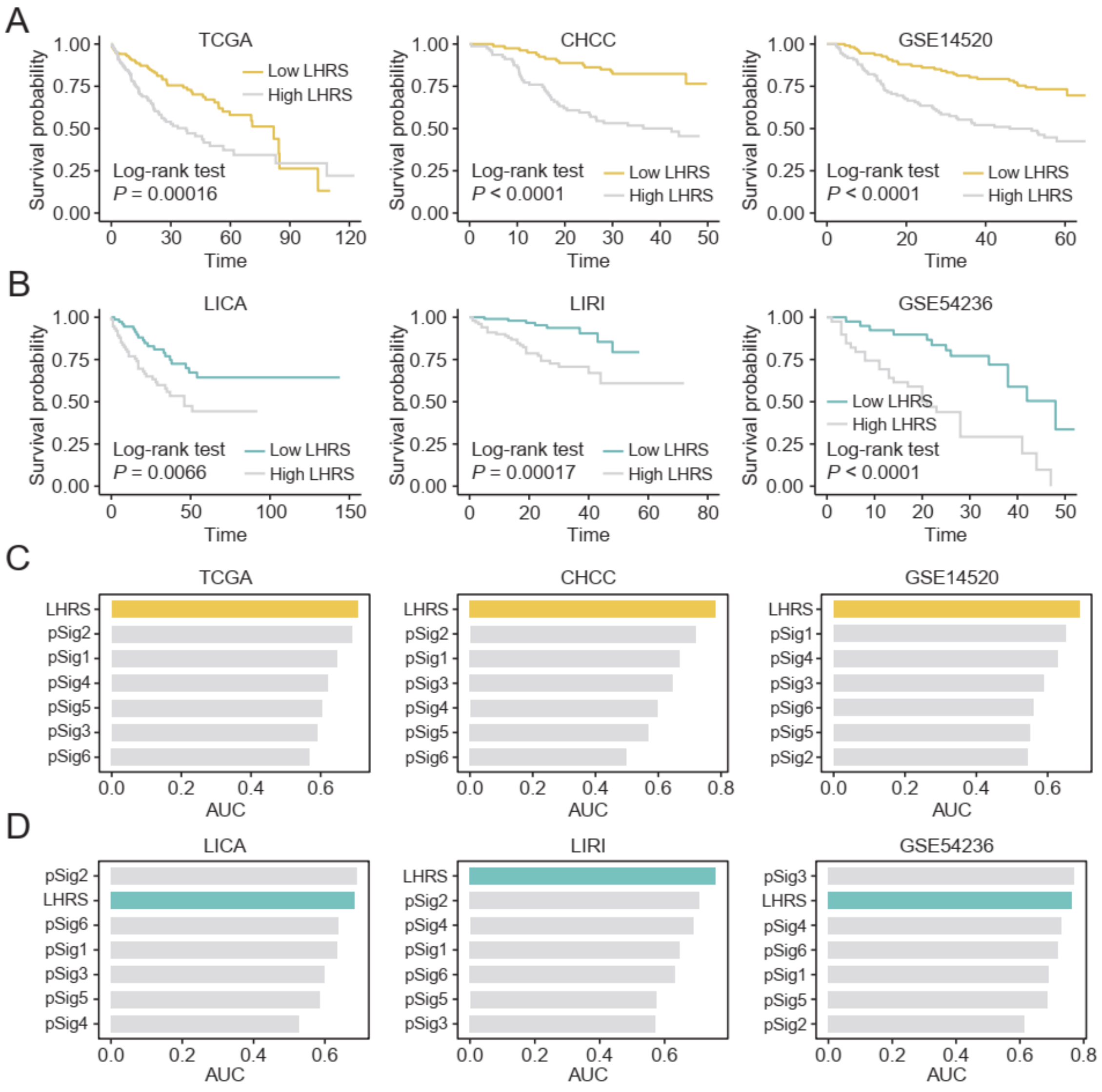

Figure S16. Evaluation of prognostic performance of LHRS. Kaplan–Meier curves of overall survival (OS) for patients stratified by median LHRS score in three training cohorts (A) and three independent testing cohorts (B). Survival difference was compared using log-rank test. Comparison of time-dependent area under the receiver operating characteristic (AUC) values (data are presented as the mean value of AUC at 12, 24, 36 and 48 months, and 60-month AUC was not calculated due to the limited follow-up time in CHCC and GSE54236 cohorts) between LHRS and six previously published signatures in three training cohorts (C) and three independent testing cohorts (D).

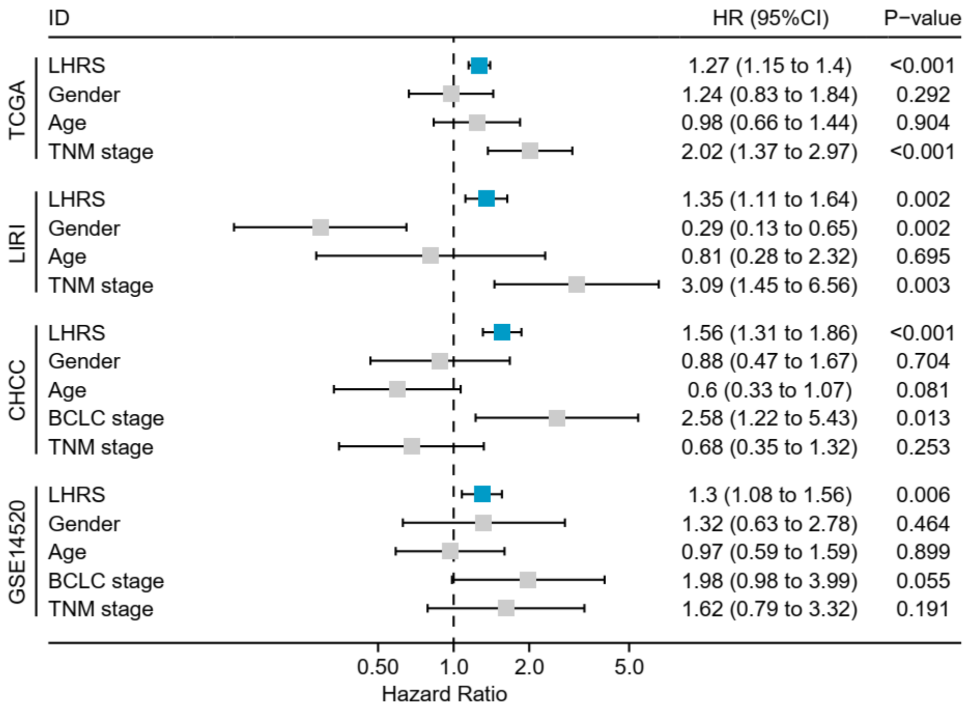

Figure S17. Prognostic value of LHRS when adjusting for gender, age, and pathological stage. Multivariate Cox regression analysis was conducted in TCGA, LIRI, CHCC and GSE14520 cohorts. Other two cohorts (LICA and GSE54236) were not included due to the deficiency of corresponding clinical information. Hazard ratios with 95% confidence interval (CI) are shown for each variables and are plotted on the natural log scale.

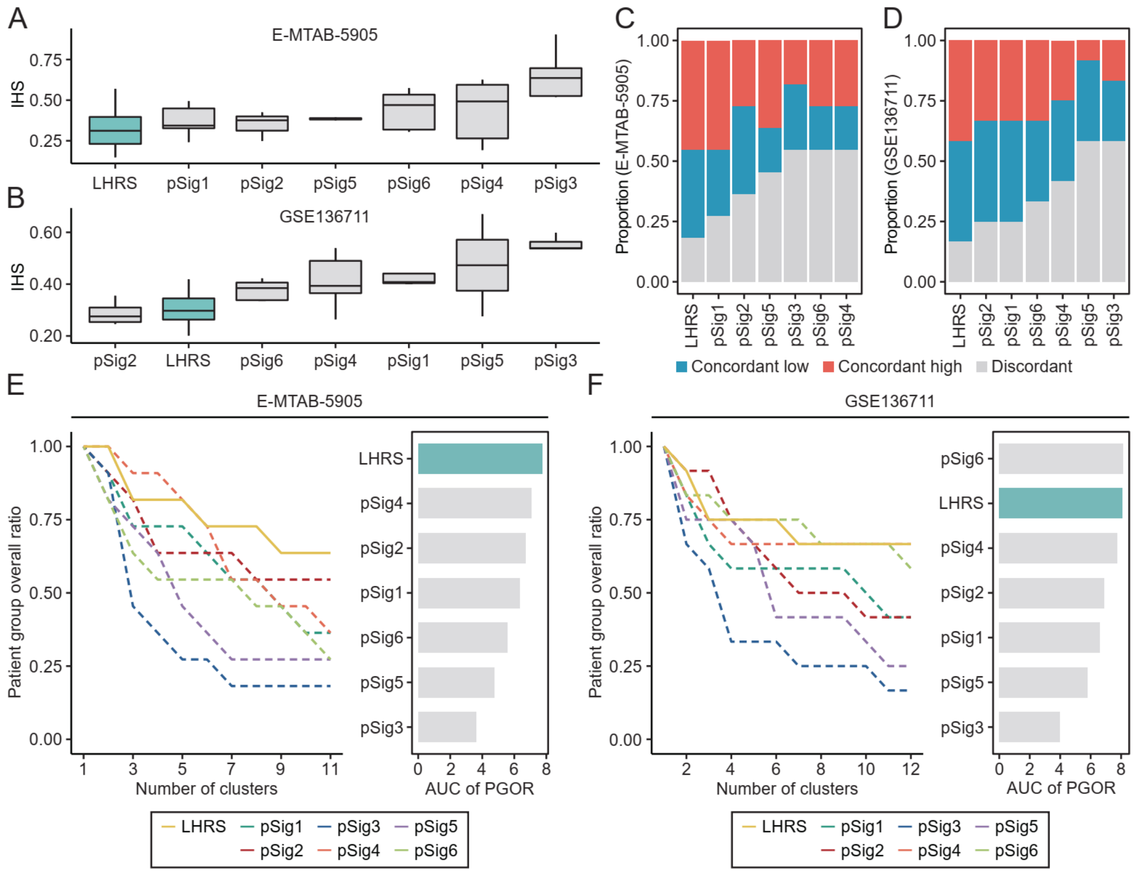

Figure S18. Evaluation of the ability to overcome intratumor heterogeneity of LHRS. Comparison of IHS of signature genes calculated on E-MTAB-5905 cohort (A) and GSE136711 cohort (B) between LHRS and other six published signatures. Comparison of the percentages of patients who were classified as concordant low risk (blue), concordant high risk (red) or discordant (gray) between LHRS and other six published signatures based on E-MTAB-5905 cohort (C) and GSE136711 cohort (D). Comparison of the ability of concordantly clustering patient samples between LHRS and other six published signatures based on E-MTAB-5905 (E) and GSE136711 cohort (F). The left panel in each figure shows the patient group overall ratio (PGOR) curves of different signatures; The right panel in each figure shows the area under the curve (AUC) values of each curve.

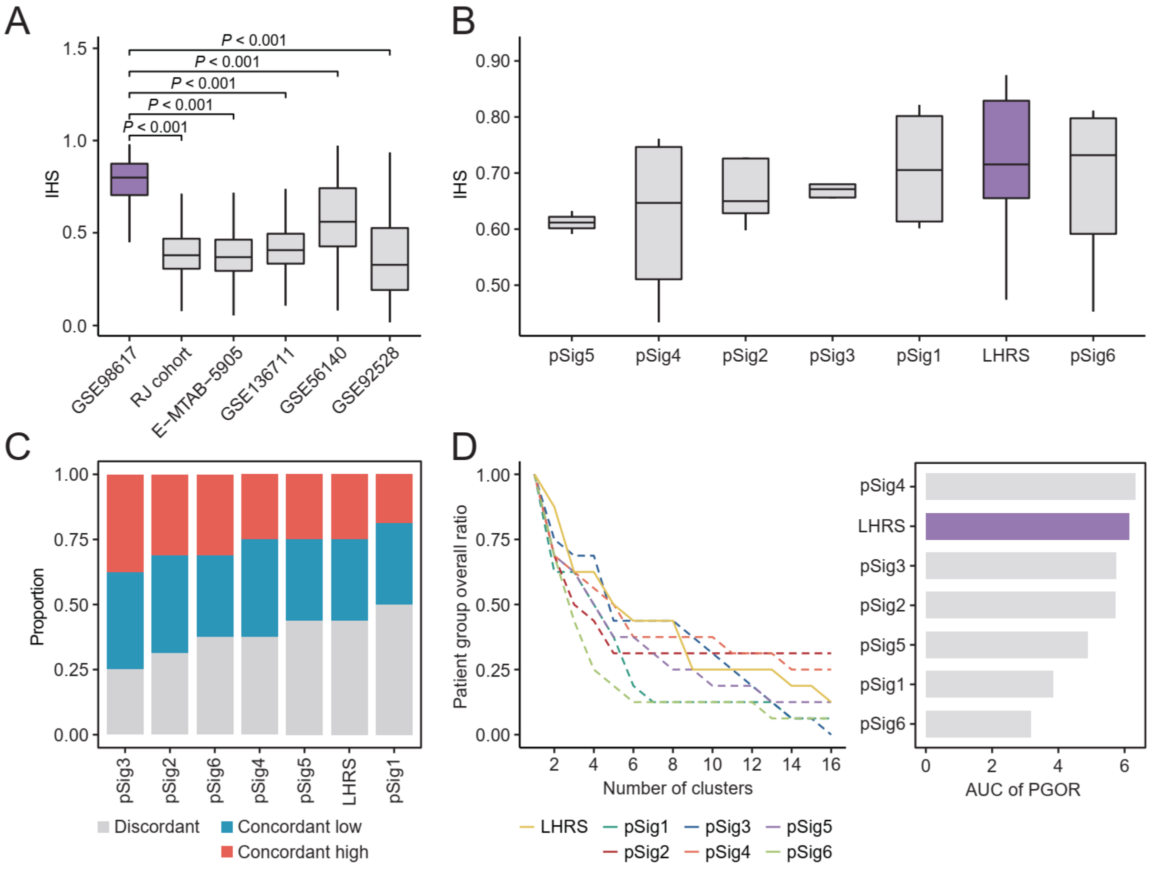

Figure S19. Evaluation of the variability of LHRS across different tumors within the same patient. (A) Comparison of IHS of genes between one multi-focal sequencing cohort (GSE98617) and five multi-region sequencing cohorts. Statistical significance of difference was determined using Wilcoxon rank-sum test. (B) Comparison of IHS of signature genes between LHRS and other six published signatures based on GSE98617 cohort. (C) Comparison of the percentages of patients who were classified as concordant low risk (blue), concordant high risk (red) or discordant (gray) between LHRS and other six published signatures based on GSE98617 cohort. (D) Comparison of the ability of concordantly clustering patient samples between LHRS and other six published signatures based on GSE98617 cohort. The left panel in each figure shows the patient group overall ratio (PGOR) curves of different signatures; The right panel in each figure shows the area under the curve (AUC) values of each curve.

References

1. Li M, Zhang Z, Li L.Wang X. An algorithm to quantify intratumor heterogeneity based on alterations of gene expression profiles. *Commun Biol.* 2020;**3**:505.
2. Ma L, Hernandez MO, Zhao Y, Mehta M, Tran B, Kelly M, et al. Tumor Cell Biodiversity Drives Microenvironmental Reprogramming in Liver Cancer. *Cancer Cell.* 2019;**36**:418-430.e6.
