# Supplementary material for: Multi-region sequencing with spatial information enables accurate heterogeneity estimation and risk stratification in liver cancer
Source: Genome Med. 2022 Dec 16;14:142. doi: 10.1186/s13073-022-01143-6 (PMC9758830; doi:10.1186/s13073-022-01143-6)
Supplement: Supplementary file 2 — Additional file 2. Supplementary Methods. [file 13073_2022_1143_MOESM2_ESM.docx]

**Supplementary Methods**

**RNA sequencing**

Total RNA was extracted from the frozen tissues by RNA mini kit (Qiagen, Hilden, Germany). Poly-A enriched RNA-seq libraries were generated using Stranded mRNA-seq kit (NOVIZAN, China) according to the manufacturer’s recommendations. RNA sequencing was performed on Illumina NovaSeq 6000 platform with 40M pair-end 150bp reads per sample. The raw reads were mapped to hg38 reference genome using HISAT2 (version 2.2.1) with default parameters [1]. Gene-level read counts were then calculated using the subRead package [2]. The resultant count data was normalized to transcript per million (TPM) for downstream analyses. Raw counts were only used for differential expression analysis based on edgeR package [3].

**Whole-exome sequencing**

For patient T10, T13, and T18, genomic DNA and total RNA were extracted simultaneously using AllPrep DNA/RNA mini kit (Qiagen, Hilden, Germany). DNA libraries were prepared using the SureSelectXT Human All Exon v4 kit (Agilent, CA, USA) following manufacturer’s instructions. Paired end, 150bp read-length sequencing was then performed on Illumina NovaSeq 6000 platform with a mean sequencing coverage of 100X. Reads in FASTQ format were aligned to hg38 reference genome using BWA-MEM (version 0.7.17-r1188) [4]. Based on GATK Best Practice pipeline (<https://software.broadinstitute.org/gatk/best-practices/>), aligned reads were further processed. Duplicate reads derived from PCR were marked by Picard tool (version 2.25.1) and base quality score was corrected by BaseRecalibrator (GATK 4.1.4). Mutect2 (GATK 4.1.4) was then used to discern single nucleotide variants (SNVs) as well as insertions and deletions (indels) using matched liver tissues as germline reference [5]. The resultant variants were annotated by Funcotator (GATK 4.1.4).

**RNA-based somatic mutation calling**

The feasibility of RNA-based mutation calling has been demonstrated by numerous studies [6-12]. In the condition of identifying SNVs for expressed genes, RNA-seq can be advantageous compared to DNA sequencing, since RNA-seq enriches for expressed transcripts and thus may increase the power to detect functionally important SNVs [6]. A recent study also reported that RNA-based SNVs were more likely to be validated at the protein level than DNA-based SNVs [12]. To achieve relatively robust variant detection, 40M pair-end 150bp reads were generated per library for RNA-seq in this study, the library size of which was twice the size of the standard library for differential expression detection (usually 20M) [13]. According to previous publications, we designed a pipeline to discern potential SNVs (**Additional file 1: Fig. S4A**). Specifically, raw tumor and normal RNA reads were first aligned using STAR (version 2.7.8a) with 2-pass mapping mode to the hg38 human reference genome [14]. Then, Picard tool (version 2.25.1) was utilized to mark PCR duplicates, SplitNCigarReads (part of GATK 4.1.4) was used to split reads spanning different exons, and BaseRecalibrator was used to correct the base quality score. The resultant 21 normal RNA-seq BAM files were used to create a Panel of Normals (PoN) by running Mutect2 in tumor-only mode and applying CreateSomaticPanelOfNormals (GATK 4.1.4), which could filter out method-specific artefacts.

Previous studies also suggested that using matched-normal DNA data for germline variant filtering could improve the detection accuracy of RNA mutation [8, 15]. Therefore, WES was performed on 10 matched-normal samples to obtain the matched germline variant profiles. Raw DNA reads were mapping to the hg38 human reference genome by BWA-MEM (version 0.7.17-r1188) [4]. Picard tool (version 2.25.1) and BaseRecalibrator were then applied to the mapped reads to obtain analysis-ready DNA BAM files. Based on tumor RNA BAM, matched normal DNA BAM, and a normal RNA PoN, Mutect2 and Funcotator were used to call and annotate mutations. Subsequently, several filtering steps were applied to further enrich for somatic variants: (1) we excluded variants in non-coding regions due to the low sequencing coverage in these areas; (2) indels were excluded due to the intrinsic technical issues of RNA-seq; (3) variants annotated as RNA-editing sites in the Rigorously Annotated Database of A-to-I RNA editing (RADAR) database (version 2) [16] or the Database of RNA editing (DARNED) database [17] were excluded; (4) variants with variant allele frequency (VAF) greater than 0.7 were excluded due to potential RNA-specific bias [9]; (5) variants located in immunoglobulin and HLA locus were also excluded since these highly polymorphic regions can be potential source of false-positive mutation calls [9]. Notably, tumors derived from patients without matched normal samples (including T02, T05, T07 and T08) were not amenable to somatic mutation calling and thus were excluded from this analysis.

**Phylogenetic tree construction**

For the construction of RNA-based and DNA-based phylogenetic trees, normalized expression data and dichotomized mutation matrix (1 for present mutation and 0 for absent mutation) were taken as input. Transcriptomic and genetic distance matrices were first calculated using the Manhattan distance. Trees were then inferred through using the neighbor-joining method in ape R package [18]. Inferred trees were recolored and the branch lengths were proportional to the transcriptomic and genetic distance.

**Prognostic signature construction**

Six HCC cohorts with available survival information were used to construct a prognostic model. Three of them, including LIHC, CHCC and GSE14520, were randomly selected as training cohorts, while the rest of them were taken as independent testing cohorts. A preliminary filtering was performed to include clonally expressed genes with low IHS (<0.25). Then, univariate Cox proportional hazards regression (COXPH) analysis was carried out using clonally expressed genes on three training cohorts respectively. Genes with significant prognostic association (*P* < 0.05) and concordant algebraic sign (protective gene has negative log2HazardRatio while risk gene has positive log2HazardRatio) in all three cohorts was retained. A bootstrapping-based approach was adopted to select genes with robust prognostic associations. Specifically, simple random sampling without replacement (SRSWOR) was conducted to extract 70% samples from the entire cohort for performing univariate COXPH analysis, which was repeated 1,000 times in each training cohort (thus leading to a total of 3,000 iterations). Genes with significant prognostic value in at least 2,000 iterations were kept for subsequent analysis.

Next, we adopted random survival forest (RSF) analysis to further narrow down the gene panel [19]. Training on LIHC cohort, the RSF analysis based on variable hunting algorithm was independently performed 1,000 times using randomForestSRC R package. The resultant gene signature in each iteration was applied to calculate the mean value of area under the receiver operating characteristic curve (AUC) at 12, 24, 36 and 48 months (60-month AUC was not calculated due to the limited follow-up time in CHCC and GSE54236 cohorts) in each training cohort. We further computed the overall mean value (AUC_μ_) and the variance (AUC_σ2_) of each signature across three training cohorts. Signatures with unstable performance (AUC_σ2_ > median value) were first filtered out. Of the remaining signature, the one with the largest AUC_μ_ was considered the optimal prognostic signature. Based on this, we defined the low-heterogeneity risk stratification (LHRS) score as follows:

LHRS score = $\frac{1}{Number(R)}\sum Exp(Risk genes)-\frac{1}{Number(P)}\sum Exp(Protective genes)$

Conceptually, HCC patients who have higher LHRS scores are more likely to experience unfavorable prognosis. To demonstrate the superiority of LHRS for prognostication in HCC, six previously published signatures, including three classic signatures (pSig1 [20], pSig2 [21], and pSig3 [22]) and three recently published signatures (pSig4 [23], pSig5 [24], and pSig6 [25]), were collected from corresponding publications for comparisons.

**Pathway enrichment analysis**

For the identification of differential biological processes between ITH-high and -low patients, we conducted gene set enrichment analysis (GSEA) using the clusterProfiler R package based on the KEGG pathway gene sets with the default parameters [26]. Leveraging KEGG pathway metadata from KEGG Pathway Maps ([www.kegg.jp/kegg-bin/get_htext?br08901.keg](http://www.kegg.jp/kegg-bin/get_htext?br08901.keg)), original gene sets were collapsed into hierarchical KEGG pathways (3 levels), which could be used to further cluster the results of enrichment analysis. For the identification of differential processes between IHS-high and -low genes, we conducted GSEA-based EnrichmentMap analysis [27, 28]. The latest GSEA desktop software (version 4.1.0) was first downloaded from the GSEA web server (<http://www.gsea-msigdb.org/gsea/downloads.jsp>). GSEA analysis was performed using the gene sets corresponding to biological processes of Gene Ontology (GO) [29] and pathways in Reactome database [30], which were obtained from g:Profiler database (<https://biit.cs.ut.ee/gprofiler/gost>) [31]. Gene sets with a size ranging from 15 and 500 were used for analysis and only gene sets that were enriched with a false discovery rate (FDR) lower than 5% were kept for subsequent analysis. EnrichmentMap analysis was conducted on the GSEA results using EnrichmentMap application in Cytoscape software (version 3.8.2) [32]. AutoAnnotate application was applied to cluster similar processes into the same groups [33]. The major biological themes of each group were extracted manually and labelled in the EnrichmentMap result.

**Functional similarity analysis**

Functional similarity analysis was utilized to clarify the correlation between LHRS genes. Functional similarity (FS) scores were calculated using the semantic similarities in molecular function (MF) and cellular component (CC) from the gene ontology (GO) terms, which takes both function and location into account [34]. Functional similarity scores was defined as follows:

FS score = $\sqrt{SimMF*SimCC}$

where semantic similarities in MF (SimMF) and CC (SimCC) were measured based on the GO topological structure through using *GOSemSim* package [35]. Gene interactions with FS scores > 0.4 were kept for the construction of interaction network using Cytoscape software [32].

**Clustering analysis**

Hierarchical clustering was performed using the top 500 genes with maximum variation on all the 96 samples (75 tumor and 21 adjacent liver samples) with RNA-seq data. T-distributed stochastic neighbor embedding (t-SNE) analysis was also conducted using Rtsne R package with the parameters of 1,000 iterations and a perplexity of 5 to generate t-SNE map of all the included samples for visualization.

**Immune feature analysis**

To estimate the burden of tumor-infiltrating lymphocyte (TIL) in each bulk sample, TRUST4 algorithm was first adopted to extract T and B cell receptor (TCR/BCR) reads that were mapped to V(D)J loci from bulk RNA-seq data [36]. Then, the TIL burden in each bulk sample was estimated using the number of V(D)J reads normalized by the total number of sequencing reads [37]. The proportions of 22 immune cell types were estimated based on bulk data using CIBERSORT algorithm (version 1.03) [38]. The LM22 basis matrix was downloaded from the CIBERSORT website (<https://cibersort.stanford.edu/>) for deconvolution. CIBERSORT was run for 1000 permutations. The resultant immune cell proportions were then used for subsequent analyses. Immune and stromal gene lists (141 immune genes and 141 stromal genes) were obtained from previous publication [39]. To determine the immune-level heterogeneity within a given tumor, we calculated the centroid-based Mahalanobis distance based on the first five PCs from the expression profiles of 141 immune genes. A threshold of Mahalanobis distance to define the outliers was determined via calculating Chi-Square value with 5 degrees of freedom (based on the number of PCAs we selected) and 0.99 probability. According to the previously defined criteria, if Mahalanobis distance of all regions within a tumor does not exceed this threshold, then this tumor is considered to have ‘concordant intra-tumor immune profiles’; otherwise, this tumor is considered to have ‘discordant intra-tumor immune profiles’ [40]. In the case of an invertible covariance matrix, a pseudo-inverse was calculated to infer Mahalanobis distance using MASS R package. Genes associated with interferon-γ (IFN-γ) response were obtained from the Molecular Signatures Database v7.5.1. A total of 36 well-established checkpoint genes (including co-inhibitory and co-stimulatory checkpoint genes) were collected from previous publications [41-43].

**Regulatory network inference**

Before the inference of protein activity, regulatory network for each protein needs first to be established. The Algorithm for the Reconstruction of Accurate Cellular Networks (ARACNe) was thus adopted to achieve this objective [44]. ARACNe analysis is only limited to ~7000 regulatory proteins, including transcription factors (GO:0003700, GO:0003677, GO:0030528, GO:0003677 or GO:0045449), transcriptional cofactors (GO:0003712, GO:0030528 or GO:0045449), signaling pathway related genes (GO:0007165, GO:0005622 or GO:0005886) and surface marker genes (GO:0005886 or GO:0009986), which have biologically meaningful downstream regulatory targets [45]. Other proteins without known signaling or transcriptional activity are not considered since their activity could be difficult to interpret biologically. ARACNe requires at least 100 samples to ensure reliable results, and thus it was only run on five public HCC cohorts included in this study (TCGA, LIRI, LICA, CHCC and GSE14520), with 100 bootstrap iterations in each cohort. Parameters of ARACNe were set to MI (Mutual Information) p-value threshold of 10^−8^ and 0 DPI (Data Processing Inequality) tolerance.

**Protein activity inference**

Weighted VIPER, an improved version of MetaVIPER, was utilized to infer the protein activity of 75 multiregional samples based on ARACNe networks from five HCC cohorts [46]. Proteins with less than 25 targets inferred by ARACNe were excluded. VIPER analysis resulted in 5,099 proteins with successfully inferred activity across all 75 samples in our cohort. Of note, only 21 out of 36 checkpoint genes (including 9 co-inhibitory and 12 co-stimulatory genes) have available protein activity inferred by VIPER.

**Differential analysis**

The differential gene expression analysis between low-ITH and high-ITH tumors was conducted using edgeR package based on the raw count data. After excluding genes that were not expressed in more than 30% of samples, 15,490 genes were retained for the differential expression analysis. The upregulated genes were defined as genes with FDR < 0.05 and log2FoldChange > 0; downregulated genes were defined as genes with FDR < 0.05 and log2FoldChange < 0. For differential protein activity analysis, limma R package was adopted. Differential proteins were defined as proteins with FDR < 0.05.

**Robust rank aggregation**

Robust Rank Aggregation (RRA) method was used to integrate the IHS results of immune cell types from different tumor types. This method has been embedded in the RobustRankAggreg R package [47]. In our case, A lower p value of certain cell type indicates an overall higher IHS value, which represent higher heterogeneity within tumors.

**Immunohistochemistry**

Immunohistochemistry for CD45 was conducted to evaluate the tumor lymphocyte infiltration according to the standard procedures. Anti-human CD45 antibody (GB14038) was purchased from Servicebio (Wuhan, China). Immunohistochemistry slides were digitally scanned using the Aperio CS Scanscope (Aperio Technologies, CA, USA) and visualized using NDP View v2 software (Hamamatsu Photonics, Japan). The density of CD45+ cell infiltration was manually calculated using three randomly chosen 20X fields. Notably, IHC was not performed on tumor T02 (n = 6) and T05 (n = 4) since their corresponding paraffin-embedded tissues were damaged during transportation (H&E slides were still available).

**References**

1. Kim D, Paggi JM, Park C, Bennett C.Salzberg SL. Graph-based genome alignment and genotyping with HISAT2 and HISAT-genotype. *Nat Biotechnol.* 2019;**37**:907-915.

2. Liao Y, Smyth GK.Shi W. The Subread aligner: fast, accurate and scalable read mapping by seed-and-vote. *Nucleic Acids Res.* 2013;**41**:e108.

3. Robinson MD, McCarthy DJ.Smyth GK. edgeR: a Bioconductor package for differential expression analysis of digital gene expression data. *Bioinformatics.* 2010;**26**:139-40.

4. Li H.Durbin R. Fast and accurate short read alignment with Burrows-Wheeler transform. *Bioinformatics.* 2009;**25**:1754-60.

5. Cibulskis K, Lawrence MS, Carter SL, Sivachenko A, Jaffe D, Sougnez C, et al. Sensitive detection of somatic point mutations in impure and heterogeneous cancer samples. *Nat Biotechnol.* 2013;**31**:213-9.

6. Piskol R, Ramaswami G.Li JB. Reliable identification of genomic variants from RNA-seq data. *Am J Hum Genet.* 2013;**93**:641-51.

7. Losic B, Craig AJ, Villacorta-Martin C, Martins-Filho SN, Akers N, Chen X, et al. Intratumoral heterogeneity and clonal evolution in liver cancer. *Nat Commun.* 2020;**11**:291.

8. Yizhak K, Aguet F, Kim J, Hess JM, Kübler K, Grimsby J, et al. RNA sequence analysis reveals macroscopic somatic clonal expansion across normal tissues. *Science.* 2019;**364**.

9. García-Nieto PE, Morrison AJ.Fraser HB. The somatic mutation landscape of the human body. *Genome Biol.* 2019;**20**:298.

10. Muyas F, Zapata L, Guigó R.Ossowski S. The rate and spectrum of mosaic mutations during embryogenesis revealed by RNA sequencing of 49 tissues. *Genome Med.* 2020;**12**:49.

11. Lindskrog SV, Prip F, Lamy P, Taber A, Groeneveld CS, Birkenkamp-Demtröder K, et al. An integrated multi-omics analysis identifies prognostic molecular subtypes of non-muscle-invasive bladder cancer. *Nat Commun.* 2021;**12**:2301.

12. Zhang Q, Lou Y, Yang J, Wang J, Feng J, Zhao Y, et al. Integrated multiomic analysis reveals comprehensive tumour heterogeneity and novel immunophenotypic classification in hepatocellular carcinomas. *Gut.* 2019;**68**:2019-2031.

13. Quaglieri A, Flensburg C, Speed TP.Majewski IJ. Finding a suitable library size to call variants in RNA-Seq. *BMC Bioinformatics.* 2020;**21**:553.

14. Dobin A, Davis CA, Schlesinger F, Drenkow J, Zaleski C, Jha S, et al. STAR: ultrafast universal RNA-seq aligner. *Bioinformatics.* 2013;**29**:15-21.

15. Hashimoto S, Noguchi E, Bando H, Miyadera H, Morii W, Nakamura T, et al. Neoantigen prediction in human breast cancer using RNA sequencing data. *Cancer Sci.* 2021;**112**:465-475.

16. Ramaswami G.Li JB. RADAR: a rigorously annotated database of A-to-I RNA editing. *Nucleic Acids Res.* 2014;**42**:D109-13.

17. Kiran A.Baranov PV. DARNED: a DAtabase of RNa EDiting in humans. *Bioinformatics.* 2010;**26**:1772-6.

18. Paradis E, Claude J.Strimmer K. APE: Analyses of Phylogenetics and Evolution in R language. *Bioinformatics.* 2004;**20**:289-90.

19. Chen X.Ishwaran H. Random forests for genomic data analysis. *Genomics.* 2012;**99**:323-9.

20. van Malenstein H, Gevaert O, Libbrecht L, Daemen A, Allemeersch J, Nevens F, et al. A seven-gene set associated with chronic hypoxia of prognostic importance in hepatocellular carcinoma. *Clin Cancer Res.* 2010;**16**:4278-88.

21. Nault JC, De Reyniès A, Villanueva A, Calderaro J, Rebouissou S, Couchy G, et al. A hepatocellular carcinoma 5-gene score associated with survival of patients after liver resection. *Gastroenterology.* 2013;**145**:176-187.

22. Villa E, Critelli R, Lei B, Marzocchi G, Cammà C, Giannelli G, et al. Neoangiogenesis-related genes are hallmarks of fast-growing hepatocellular carcinomas and worst survival. Results from a prospective study. *Gut.* 2016;**65**:861-9.

23. Fang Q.Chen H. Development of a Novel Autophagy-Related Prognostic Signature and Nomogram for Hepatocellular Carcinoma. *Front Oncol.* 2020;**10**:591356.

24. Fang Q.Chen H. The significance of m6A RNA methylation regulators in predicting the prognosis and clinical course of HBV-related hepatocellular carcinoma. *Mol Med.* 2020;**26**:60.

25. Pan Q, Qin F, Yuan H, He B, Yang N, Zhang Y, et al. Normal tissue adjacent to tumor expression profile analysis developed and validated a prognostic model based on Hippo-related genes in hepatocellular carcinoma. *Cancer Med.* 2021;**10**:3139-3152.

26. Wu T, Hu E, Xu S, Chen M, Guo P, Dai Z, et al. clusterProfiler 4.0: A universal enrichment tool for interpreting omics data. *Innovation (N Y).* 2021;**2**:100141.

27. Merico D, Isserlin R, Stueker O, Emili A.Bader GD. Enrichment map: a network-based method for gene-set enrichment visualization and interpretation. *PLoS One.* 2010;**5**:e13984.

28. Reimand J, Isserlin R, Voisin V, Kucera M, Tannus-Lopes C, Rostamianfar A, et al. Pathway enrichment analysis and visualization of omics data using g:Profiler, GSEA, Cytoscape and EnrichmentMap. *Nat Protoc.* 2019;**14**:482-517.

29. Ashburner M, Ball CA, Blake JA, Botstein D, Butler H, Cherry JM, et al. Gene ontology: tool for the unification of biology. The Gene Ontology Consortium. *Nat Genet.* 2000;**25**:25-9.

30. Fabregat A, Jupe S, Matthews L, Sidiropoulos K, Gillespie M, Garapati P, et al. The Reactome Pathway Knowledgebase. *Nucleic Acids Res.* 2018;**46**:D649-d655.

31. Raudvere U, Kolberg L, Kuzmin I, Arak T, Adler P, Peterson H, et al. g:Profiler: a web server for functional enrichment analysis and conversions of gene lists (2019 update). *Nucleic Acids Res.* 2019;**47**:W191-w198.

32. Cline MS, Smoot M, Cerami E, Kuchinsky A, Landys N, Workman C, et al. Integration of biological networks and gene expression data using Cytoscape. *Nat Protoc.* 2007;**2**:2366-82.

33. Kucera M, Isserlin R, Arkhangorodsky A.Bader GD. AutoAnnotate: A Cytoscape app for summarizing networks with semantic annotations. *F1000Res.* 2016;**5**:1717.

34. Han Y, Yu G, Sarioglu H, Caballero-Martinez A, Schlott F, Ueffing M, et al. Proteomic investigation of the interactome of FMNL1 in hematopoietic cells unveils a role in calcium-dependent membrane plasticity. *J Proteomics.* 2013;**78**:72-82.

35. Yu G, Li F, Qin Y, Bo X, Wu Y.Wang S. GOSemSim: an R package for measuring semantic similarity among GO terms and gene products. *Bioinformatics.* 2010;**26**:976-8.

36. Song L, Cohen D, Ouyang Z, Cao Y, Hu X.Liu XS. TRUST4: immune repertoire reconstruction from bulk and single-cell RNA-seq data. *Nat Methods.* 2021;**18**:627-630.

37. Zhang J, Hu X, Wang J, Sahu AD, Cohen D, Song L, et al. Immune receptor repertoires in pediatric and adult acute myeloid leukemia. *Genome Med.* 2019;**11**:73.

38. Newman AM, Liu CL, Green MR, Gentles AJ, Feng W, Xu Y, et al. Robust enumeration of cell subsets from tissue expression profiles. *Nat Methods.* 2015;**12**:453-7.

39. Yoshihara K, Shahmoradgoli M, Martínez E, Vegesna R, Kim H, Torres-Garcia W, et al. Inferring tumour purity and stromal and immune cell admixture from expression data. *Nat Commun.* 2013;**4**:2612.

40. Shen YC, Hsu CL, Jeng YM, Ho MC, Ho CM, Yeh CP, et al. Reliability of a single-region sample to evaluate tumor immune microenvironment in hepatocellular carcinoma. *J Hepatol.* 2020;**72**:489-497.

41. Charoentong P, Finotello F, Angelova M, Mayer C, Efremova M, Rieder D, et al. Pan-cancer Immunogenomic Analyses Reveal Genotype-Immunophenotype Relationships and Predictors of Response to Checkpoint Blockade. *Cell Rep.* 2017;**18**:248-262.

42. Qin S, Xu L, Yi M, Yu S, Wu K.Luo S. Novel immune checkpoint targets: moving beyond PD-1 and CTLA-4. *Mol Cancer.* 2019;**18**:155.

43. Marin-Acevedo JA, Kimbrough EO.Lou Y. Next generation of immune checkpoint inhibitors and beyond. *J Hematol Oncol.* 2021;**14**:45.

44. Lachmann A, Giorgi FM, Lopez G.Califano A. ARACNe-AP: gene network reverse engineering through adaptive partitioning inference of mutual information. *Bioinformatics.* 2016;**32**:2233-5.

45. Obradovic A, Chowdhury N, Haake SM, Ager C, Wang V, Vlahos L, et al. Single-cell protein activity analysis identifies recurrence-associated renal tumor macrophages. *Cell.* 2021;**184**:2988-3005.e16.

46. Obradovic A, Vlahos L, Laise P, Worley J, Tan X, Wang A, et al. PISCES: A pipeline for the Systematic, Protein Activity-based Analysis of Single Cell RNA Sequencing Data. *bioRxiv.* 2021:2021.05.20.445002.

47. Kolde R, Laur S, Adler P.Vilo J. Robust rank aggregation for gene list integration and meta-analysis. *Bioinformatics.* 2012;**28**:573-80.
